# T1- and diffusion tensor-based fractal dimension of white and grey matter in multiple sclerosis

**DOI:** 10.3389/fneur.2025.1618319

**Published:** 2026-01-07

**Authors:** Weronika Mazur-Rosmus, Zofia Schneider, Agnieszka Słowik, Artur Tadeusz Krzyżak

**Affiliations:** 1 LaTiS NMR Tomography and Spectroscopy Laboratory, Department of Fossil Fuels, Faculty of Geology, Geophysics and Environmental Protection, AGH University of Krakow, Krakow, Poland; 2 Department of Neurology, Jagiellonian University Medical College, University Hospital in Krakow, Krakow, Poland

**Keywords:** diffusion tensor imaging, fractal dimension, fractional anisotropy, multiple sclerosis, non-uniform gradients, systematic error

## Abstract

**Introduction:**

Multiple sclerosis (MS) changes brain microstructure even at early disease stages, with changes detectable in normal-appearing white matter (NAWM) and grey matter (GM). Diffusion tensor imaging (DTI) is sensitive to such alterations, while fractal dimension (FD) provides complementary information on tissue complexity. We hypothesized that T1-based FD offers additional diagnostic value beyond DTI-derived metrics of tissue integrity, including fractional anisotropy (FA) and mean diffusivity (MD).

**Methods:**

MRI data were acquired from 120 patients with relapsing–remitting MS and low mean Expanded Disability Status Scale (EDSS) scores, as well as 75 healthy control (HC) participants. FD, FA, and MD were quantified in global brain tissues and within white matter (WM) skeletons, both with and without lesion masking. The interactions between FD and DTI metrics were assessed, and classification models were constructed to evaluate diagnostic performance.

**Results:**

WM in MS exhibited reduced FD and FA alongside elevated MD, consistent with demyelination and axonal degradation. GM demonstrated higher FD, FA, and MD values, suggesting a more nuanced interplay of inflammatory remodeling, dendritic reorganization and compensatory structural adaptation. The limited impact of lesion masking on group-average metrics, but its marked effect on FD-DTI interactions, revealed that lesions regulate structural variance without dominating global tissue complexity. FD proved particularly sensitive at tissue interfaces, where geometry is disrupted, whereas WM skeleton analyses reflected preserved regularity of core tracts, even amid microstructural degeneration.

**Discussion:**

Our findings support the concept of a surface-in gradient of complexity loss in MS. Combining FD with FA and MD substantially improved classification accuracy, particularly in WM skeleton-based models, emphasizing the diagnostic potential of geometric-microstructural integration. Widespread associations of FA with clinical covariates and age further suggest that diffuse WM alterations underpin both cognitive and clinical decline.

## 1 Introduction

Multiple sclerosis (MS) is a neurological disease characterized by inflammation, demyelination, and neurodegeneration within the central nervous system. Recent research has emphasized the heterogeneity of MS, recognizing it as a spectrum of diseases (1). Diffusion tensor imaging (DTI) is an MRI technique sensitive to molecular diffusion and capable of modeling its anisotropy to characterize the underlying microstructural properties of the medium. In MS, DTI can be used to detect and quantify brain tissue damage that is not visible on other MRI modalities (2). DTI can reveal early-stage or subtly active MS by identifying microstructural changes in normal-appearing white matter (NAWM) (3). It is also suitable for monitoring disease progression and evaluating treatment efficacy (3). Commonly used DTI metrics include mean diffusivity (MD), which reflects overall diffusivity and increases with tissue rarefaction or damage, and fractional anisotropy (FA), which decreases with loss of fiber coherence, demyelination, or other shifts toward isotropic diffusion.

Fractal dimension (FD) is a mathematical descriptor of structural complexity or irregularity that describes its roughness (4). In the context of brain anatomy, FD reflects how intricately a structure fills space. In neuroimaging, FD is often estimated using techniques like box-counting (5) and is applied to images derived from T1-weighted MRI or DTI. In grey matter (GM), FD captures surface complexity and gyrification patterns, with altered FD reflecting changed folding area, sulcal depth, and curvature (1, 6). In WM, FD reflects the spatial complexity and branching of the fiber architecture (7, 8), and reductions in WM FD, particularly when computed from anisotropy maps or skeletonized DTI representations, have been linked to demyelination, axonal loss, and diffuse disorganization (9). A more detailed review of FD applications in neurological disorders can be found elsewhere (1).

T1- and DTI-based FD are used in studies of numerous neurological diseases, such as MS (9, 10), Alzheimer’s disease (11), mild cognitive impairment (MCI) (12), schizophrenia, and other psychiatric disorders (1). In MS patients, cortical GM FD was shown to be a potential marker of a higher risk of disability accumulation (10). Other findings show increased FD in GM among MS patients compared to healthy controls, indicating early structural changes even in patients with short disease durations (13). Conversely, decreased FD in WM has been associated with diffuse damage and was influenced by the presence of plaques and microstructural changes in normal-appearing WM (9). Although FD reflects macro-scale geometric organization and DTI reflects micro-scale diffusion properties, few studies have examined how these measures relate to each other in MS. Understanding this relationship is important because microstructural disruptions detected by DTI may propagate to the macro-scale geometry reflected in FD, or FD may capture complementary aspects of structural change not reflected in FA or MD. For example, a study on patients with small vessel disease and MCI employed machine learning techniques to integrate FD and DTI features, finding that WM FD was a consistent predictor of cognitive performance (12).

In this study, we focus on global measures of FD, which provide a whole-brain description of structural complexity in GM and WM and integrate both focal and diffuse pathology without requiring *a priori* assumptions about lesion location or specific anatomical targets. Such global measures are particularly valuable for assessing whether widespread structural disruption is present, independent of regional variability. Despite increasing interest in FD, direct studies correlating FD with DTI metrics in MS, particularly in cohorts with low mean Expanded Disability Status Scale (EDSS), remain limited. Therefore, the primary objective of this study is to assess whether global FD measures provide information specific to MS that is complementary to FA and MD. To address this aim, we first evaluate whether FD differentiates MS patients from healthy controls and then quantify FD-DTI correlations to determine whether FD reflects overlapping or independent structural information relative to FA and MD. Furthermore, we examined the robustness of global FD and DTI measures to methodological choices, including lesion masking, GM/WM mask type (general structure or skeleton), and diffusion tensor estimation using either the standard constant-in-space b-matrix (sDTI) or the spatially distributed b-matrix (BSD) correction (14–16), which accounts for systematic errors caused by non-uniformity in the gradient field. By integrating global FD and DTI metrics and evaluating their sensitivity and associations, this study aims to identify multiscale structural markers that reflect MS-related pathology and to assess the stability of these measures across common methodological variations. Because global measures were computed on whole-brain GM and WM masks, lesions were initially included to capture overall structural burden. For completeness, results were also compared with results before and after lesion masking.

## 2 Methods

### 2.1 Subjects

Subjects were recruited at The University Hospital in Krakow, Poland, and involved 150 patients with MS (later called “MS” group) and 100 healthy control volunteers (“HC” group). Among them, 20 patients and 25 HC were not qualified for the project (7 MS and 14 HC) or were excluded from this study due to incomplete MRI data (23 MS and 11 HC). Ultimately, there were 120 subjects in the MS and 75 subjects in the HC. All subjects were aged 18–50 and assessed in terms of hand function (9-Hole Peg Test), level of fatigue (Fatigue Scale for Motor and Cognitive Functions), and cognitive modalities (Symbol Digit Modalities Test, SDMT). Patients were diagnosed with relapsing–remitting MS, treated with disease-modifying therapy (DMT), and additionally rated with the EDSS with an inclusion constraint of EDSS = 0–6.5. HC individuals were eligible for the study if no lesions were identified on MRI. Within the RRMS cohort, 48% of individuals were in an early stage of the disease, defined as a disease duration of less than 5 years. HC participants had no clinical symptoms or history suggestive of MS. At the time of MRI examination, most patients were treated with dimethyl fumarate (*n* = 60), while smaller groups received other platform therapies (MET) such as interferons (*n* = 18), glatiramer acetate (*n* = 14), teriflunomide (*n* = 7), fingolimod (*n* = 4), and cladribine (*n* = 3), or high-efficacy agents (HET) like natalizumab (*n* = 6) and ocrelizumab (*n* = 3). Since there were only nine patients on HET and they were aligned stochastically within the MET group (they were not outlying), these two therapies were not separated in the analyses. All procedures, including recruitment and examinations, were approved by the Bioethics Committee at the Regional Medical Chamber, No. 282/KBL/OIL/2020 of December 18, 2020, and a positive opinion for the clinical trial was issued. Descriptive statistics for groups are shown in Table 1.

**Table 1:** Descriptive statistics for study groups.

Metric	HC	MS	Group comparison
Age (Mean ± SD)	33.80 ± 8.02	36.05 ± 6.98	*p* < 0.05
EDSS (Mean ± SD)	0.00 ± 0.00	1.51 ± 1.06	NA
SDMT (Mean ± SD)	54.61 ± 8.73	55.66 ± 11.55	*p* = 0.46
Brain volume (Mean ± SD) (ml)	1,253 ± 102	1,208 ± 117	*p* < 0.001
Brain parenchymal fraction (Mean ± SD) (%)	83.3 ± 1.5	81.7 ± 2.2	*p* < 0.001
Lesion volume (Mean ± SD) (ml)	0.00 ± 0.00	9.7 ± 11.8	NA
Lesion parenchymal fraction (LPF) (Mean ± SD) (%)	0.00 ± 0.00	0.8 ± 1.0	NA

MS, multiple sclerosis patients; HC, healthy control; EDSS, expanded disability status scale score; SDMT, Symbol Digit Modalities Test score; SD, standard deviation; NA, not applicable.

### 2.2 MRI protocol

MS patients and HC volunteers were examined in a 3 T Magnetom Vida Fit scanner (Siemens, Germany). T1-weighted imaging was conducted applying magnetization-prepared rapid gradient echo (MPRAGE) pulse sequence (TR/TE = 2300/2.99 ms, field of view, FOV = 242 mm x 250 mm, voxel size 0.98 mm x 0.98 mm x 1.20 mm), while DTI applying spin-echo echo-planar imaging (SE-EPI) pulse sequence (number of diffusion gradient directions, NDGD = 20 per shell, b-values = 0, 1,000, 2000 s/mm^2^, TR/TE = 3900/88 ms, FOV = 191×191 mm^2^, voxel size 2.53 mm x 2.53 mm x 2.50 mm), T2-weighted imaging applying turbo spin echo (TSE) pulse sequence (TR/TE = 9150/80 ms, FOV = 208 mm x 230 mm, voxel size = 0.51 mm x 0.51 mm x 3.00 mm), and fluid-attenuated inversion recovery (FLAIR) using a T2-weighted SPACE sequence with inversion recovery (TR/TE/TI = 7300/426/900 ms, FOV = 250 mm x 250 mm, voxel size = 0.49 mm x 0.49 mm x 1.00 mm). DTI was also performed for a calibration phantom used in the BSD method, applying the same protocol and table position as for each individual. The BSD-DTI approach is described in the next section.

### 2.3 Data preprocessing and diffusion tensor calculations

Structural T1-weighted images were preprocessed using the *fmriprep* software (17), which includes brain extraction with ANTs, spatial normalization to MNI152 space, and tissue segmentation using FSL FAST. GM and WM probability maps generated by FAST were thresholded at 0.5 and binarized to obtain subject-specific GM and WM masks. Additionally, the SynthSeg software was used to obtain volumetric measurements from T1-weighted images. Based on these results, the brain parenchymal fraction (BPF) was calculated as the ratio of brain parenchymal volume to total intracranial volume. MS lesions were manually delineated on FLAIR images (for supratentorial lesions) and T2-weighted images (for infratentorial lesions) by a team of experienced neuroradiologists and data scientists. Subsequently, the total lesion volume was derived, and the lesion parenchymal fraction (LPF) was calculated as the ratio of lesion volume to brain volume. DTI data were denoised using the Local Principal Component Analysis (LPCA) method in DIPY (18). All diffusion-weighted images (DWI) were registered to the image without diffusion weighting, i.e., the b0 image, using affine registration in FSL software (19). Diffusion tensors were calculated: (1) using vendor-provided b-matrix (constant in space)- this approach will be called sDTI; (2) using spatially variant, b(r) matrices obtained from the BSD method- BSD approach. Diffusion tensor MD and FA maps were registered to T1 space using the following pipeline: (1) b0 images were aligned to T1 images using FLIRT (FSL) with 6 degrees of freedom; (2) alignment matrices were used to guide boundary-based registration (BBR) using FLIRT (FSL) and WM masks from *fmriprep*; (3) BBR registration matrices were inverted and applied to FA and MD maps.

#### 2.3.1 B-matrix spatial distribution calibration

The BSD-DTI approach (15, 16, 20, 21) was used to correct spatially dependent biases in diffusion gradient amplitude and directions caused by gradient nonlinearity and other hardware imperfections. Standard DTI (sDTI) assumes a single nominal B-matrix for all voxels, but in practice, it varies with position in the magnet. BSD accounts for this by estimating a voxel-wise B-matrix, *b(r)*, derived from the Generalized Stejskal-Tanner (GST) equation using a calibration phantom with known diffusion properties. The voxel-wise B-matrix, b(r) , can be defined using the magnetic field gradient transformation tensor L(r) , which describes the local deviation of the actual gradient fields from the nominal ones:


$$b(r)=L(r)b_{\mathrm{STD}}L{\left(r\right)}^{T}$$ (1)

where $b_{\mathrm{STD}}$ is the standard, constant B-matrix provided by the vendor. During tensor fitting in subject data, these spatially varying *b(r)* matrices replace the global nominal B-matrix, thereby reducing systematic, position-dependent errors in DTI metrics. Full mathematical details are provided in the original BSD papers and patents (15, 16, 20–21).

### 2.4 Tract-based spatial statistics (TBSS)

TBSS analysis (22) was conducted in FSL (19) using standard TBSS pipeline according to FSL documentation: (1) 3D FA maps were generated for each subject from 4D sDTI and BSD NiFTI files obtained from the in-house software (BSD-DTI v.2.0, AGH, Krakow, Poland) using *fslroi* utility; (2) FA maps were prepared using *tbss_1_preproc*; (3) FA maps were nonlinearly registered to a standard space using *tbss_2_reg*; (4) mean FA maps for sDTI and BSD were generated and skeletonized using *tbss_3_postreg*; (5) individual subject FA map was projected onto mean FA skeleton using *tbss_4_prestats* and commonly used threshold = 0.2 as suggested by Smith et al. (22). To evaluate the spatial robustness of significant clusters, we additionally generated negative-effect masks (MS < HC) at three FA skeleton thresholds (0.15, 0.20, 0.25). For each threshold and model, we extracted: number of significant voxels, mean FA values for MS and HC, and effect size (Cohen’s d). Spatial consistency between masks was quantified using pairwise Dice coefficients and percentage overlap relative to each mask. These metrics were aggregated into overlap matrices and used for hierarchical clustering with average linkage, yielding dendrograms that identified stable clusters across thresholds and models. All analyses in the second approach were implemented in Python (NumPy, SciPy, scikit-learn, pandas, matplotlib).

For FD analysis, two group-mean FA skeletons were generated separately for the MS and HC cohorts. These skeletons were binarized and used as input for FD estimation in MATLAB (see Section 2.5), yielding a single FD value per group. This approach provides a measure of global structural complexity along the most coherent WM tracts for each cohort. It also reduces the influence of individual variability and minor misalignments, capturing overarching patterns of microstructural organization at the group level.

In the second approach, FA maps projected onto the one common skeleton for each subject were used. FSL’s *randomise* function was then used to test statistically significant group differences in DTI metrics, with Threshold-Free Cluster Enhancement (TFCE) applied to correct for multiple comparisons across space and 5,000 permutations. Both contrasts (MS > HC and HC > MS) were included to enable two-tailed testing. TFCE-corrected *p* < 0.05 in either direction were considered significant, and directionality was assigned *post hoc* based on the sign of the t-statistics. Contrasts examined in a hierarchical model were as follows: (1) HC ≠ MS; (2) HC ≠ MS controlling for age; (3) HC ≠ MS controlling for age and SDMT; (4) HC ≠ MS controlling for age, SDMT, and EDSS.

### 2.5 Data analysis

T1-based FD was calculated for GM and WM masks obtained from *fmriprep,* and TBSS-based FD for group-wise WM skeletons. The software was shown to be robust even in the presence of lesions (17). FD calculation was performed in MATLAB (The MathWorks Inc., MATLAB Version: 23.2.0.2409890 (R2023b) Update 3, Natick, Massachusetts, USA) according to the standard approach described in Ruiz de Miras (5) using the 3D box-counting method (23). FD was calculated from the linear part of log–log box-counting (i.e., log–log dependency of the number of boxes needed to cover nonzero elements of an image and box size). Mean FA and MD were calculated: (1) in WM and GM using *fmriprep* masks (binary threshold for probabilistic masks- 0.5) in native T1 space to avoid losing details, which occurred for masks registered to native DTI space; (2) in WM skeletons in FMRIB58 space. FA and MD values were correlated with FD values. Statistical differences between the MS and HC groups were assessed using a two-sample t-test for normally distributed data and the Mann-Whitney U test for non-normally distributed data. Normality was verified using the Shapiro–Wilk test. *p*-values were corrected for false discovery rate (FDR) using the Benjamini-Hochberg (BH) method (24).

#### 2.5.1 Robustness analysis of correlations

To assess the robustness of correlations between FD and DTI metrics (FA, MD), we complemented standard Pearson correlation (delivering the correlation coefficient, *r_p_*) with Spearman correlation (*ρ*), outlier-resistant Huber regression, and leave-one-out cross-validation (LOO-CV). All analyses were performed in Python (v3.10) using pandas, numpy, scipy.stats, and sklearn.linear_model. HuberRegressor. Pearson and Spearman (*ρ*) coefficients were computed with pearsonr() and spearmanr(), while the Huber slope provided an outlier-robust estimate of the relationship. The LOO *r_CV_* value, derived from the Pearson correlation between observed and cross-validated predictions, served as an indicator of model stability. Correlations were considered robust when the direction and sign were consistent across all three approaches (Pearson, Huber, and LOO).

#### 2.5.2 Residual redundancy modeling

To assess whether FD carries diagnostically relevant information that is not redundant with classical DTI metrics (FA, MD), demographic (age), and volumetric covariates (BPF), we used linear residual modeling. We fitted an OLS regression model:


$$\mathrm{FD}=\beta_{0}+\beta_{1}\mathrm{FA}+\beta_{2}\mathrm{MD}+\beta_{3}\mathrm{AGE}+\beta_{4}\mathrm{BPF}+\epsilon$$ (2)

From the model, residuals (*ε*) were extracted as variance in FD that is not explained by FA, MD, age, and BPF. Predictor collinearity was evaluated using the Variance Inflation Factor (VIF). Next, we tested whether residual FD still distinguishes between groups. Normality assumptions were assessed using Levene’s test (homogeneity of variances) and the Welch/Student t-test in case of normal distributions, while the Mann–Whitney U test otherwise. Multiple comparison corrections were applied using BH-FDR separately for t-tests and MW tests. Distributions of group-specific residuals were visualized using density histograms (with KDE), enabling qualitative comparison of variance retention after covariate adjustment. To evaluate the robustness of between-group differences, we additionally performed bootstrap resampling (*N* = 5,000) on group mean differences of the FD residuals.

#### 2.5.3 Receiver operating characteristic curve

To evaluate the discriminative performance of MRI metrics, receiver operating characteristic (ROC) curves were constructed in a bootstrap analysis (*N* = 1,000), and the area under the curve (AUC) was calculated. Optimal cut-off values were determined using the Youden index. Sensitivity, specificity, and 95% confidence intervals for AUC were reported.

## 3 Results

**Figure 1:**
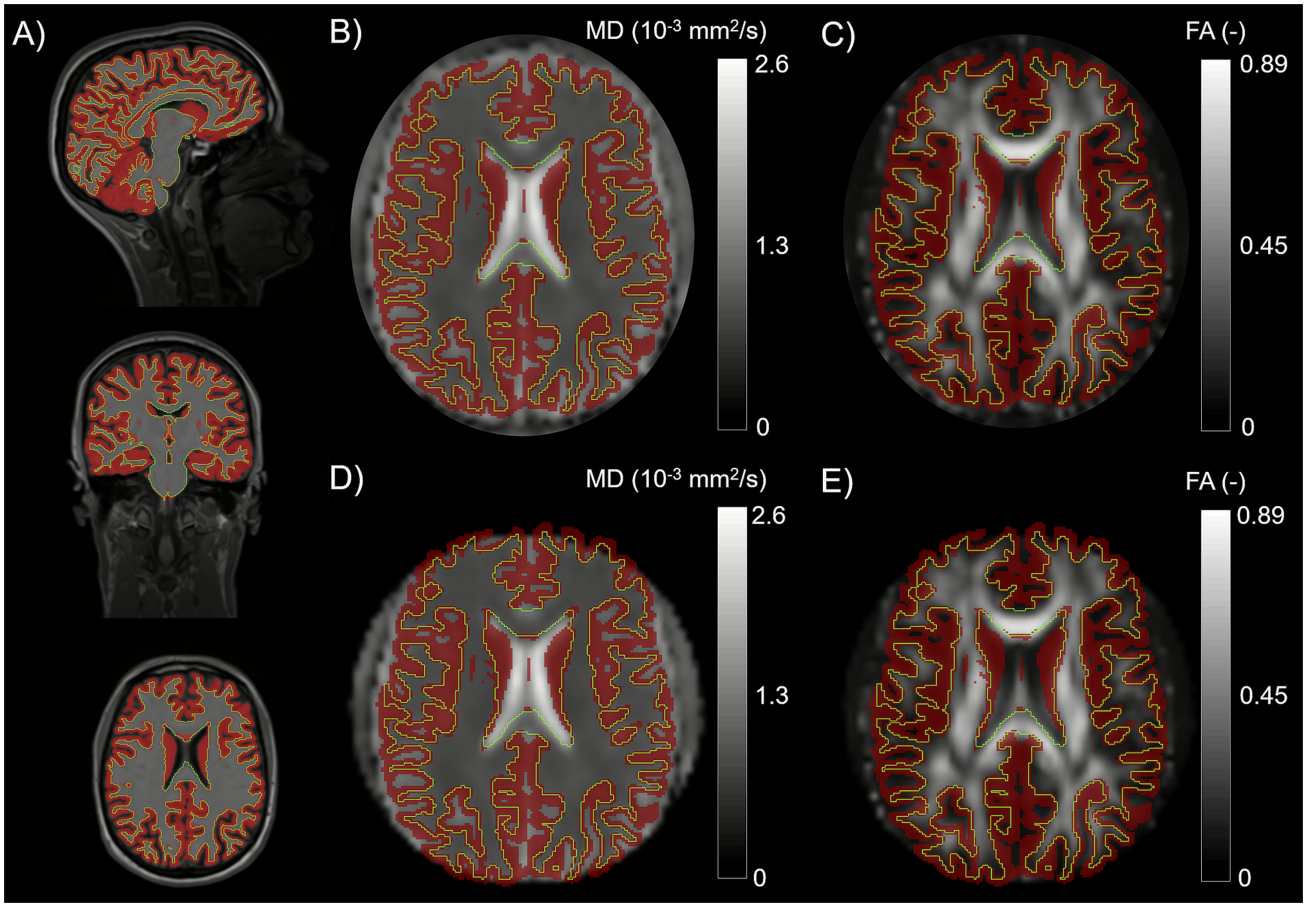
White matter mask contour (yellow contour) and grey matter mask (red overlay) for an example multiple sclerosis (MS) patient overlaid on T1 image in 3 orthogonal planes **(A)**, mean diffusivity (MD) map from standard diffusion tensor calculations approach (sDTI) **(B)** and after non-uniform magnetic field gradient calibration (BSD) **(D)**, fractional anisotropy (FA) map from sDTI **(C)** and BSD **(E)**.

FD was calculated for each subject in the WM and GM masks obtained from T1 images. Then, FA and MD maps were registered to the T1 space, and weighted mean (based on probabilistic mask weights) FA and MD were calculated in the same WM and GM masks. An example of these masks overlaid on FA and MD maps in T1 space for a representative MS patient is shown in Figure 1 for sDTI (Figures 1B,C) and BSD approaches (Figures 1D,E). Since the BSD method relies on phantom measurements to determine b-matrix maps (Equation 1), all images were additionally masked out by a phantom mask before any calculations.

### 3.1 Comparison of metrics across groups

**Figure 2:**
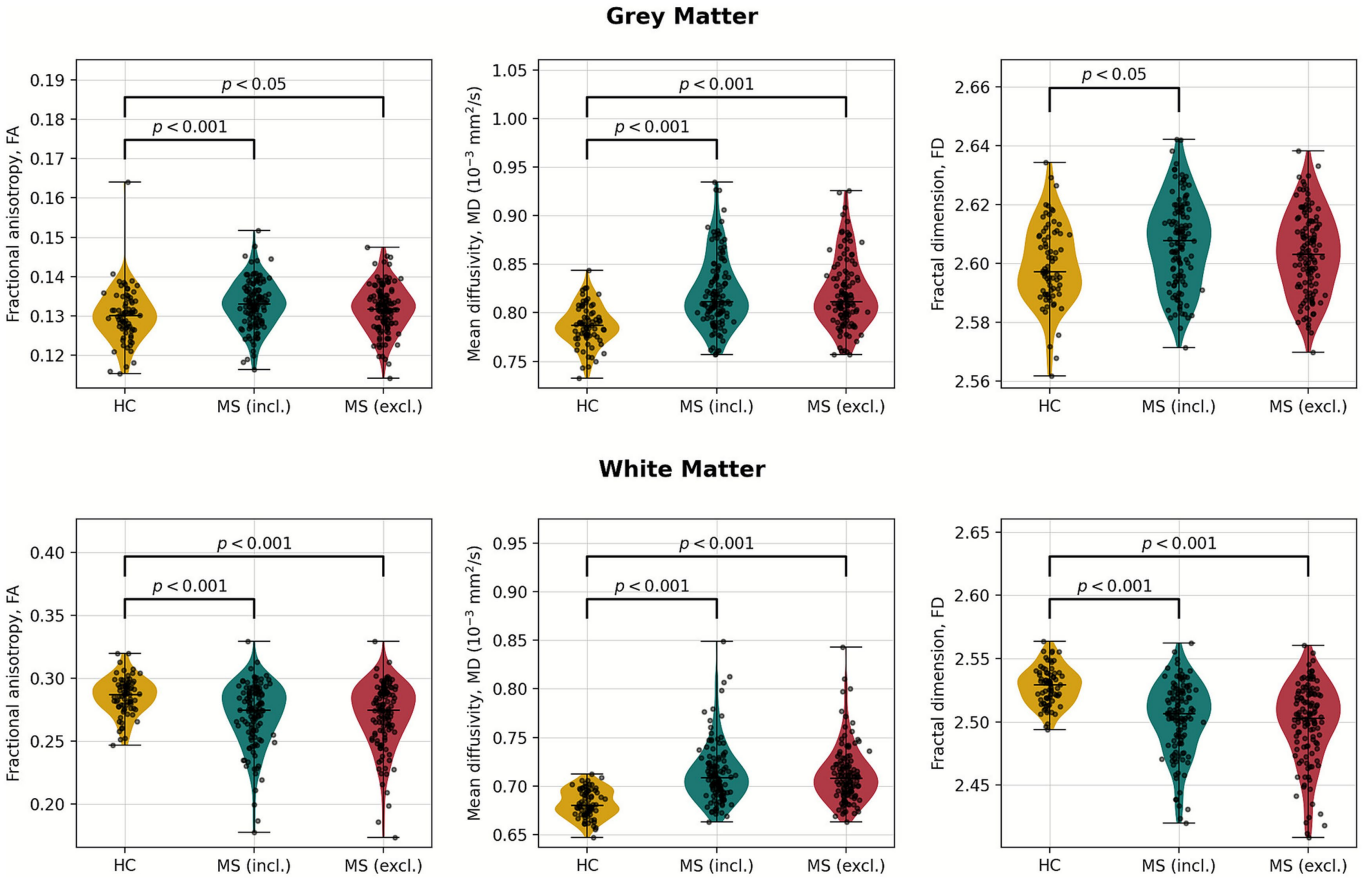
Distributions of fractional anisotropy (FA), mean diffusivity (MD), and T1-based fractal dimension (FD) in healthy controls (HC) and multiple sclerosis (MS) cohorts. Distributions for MS were analyzed without (“(incl.)”) and with (“(excl.)”) lesion masking. Brackets indicate statistically different mean values at a significance level of 0.05, with a probability located above the bracket. For FA in GM Welch, the t-test was used, whereas the Mann–Whitney U-test was used for the remaining comparisons.

The WM/GM general structure group results of FD, MD, and FA before and after lesion masking are shown in Figure 2 and summarized in Table 2 for BSD and Supplementary Table S1 for sDTI. FD was higher in GM than in WM. Mean FA values in WM were ~0.3 due to the mean calculated from the whole WM. Moreover, MS are more dispersed than HC.

**Table 2:** Mean fractional anisotropy (FA), mean diffusivity (MD), and T1-based fractal dimension (FD) in healthy controls (HC) and multiple sclerosis patients (MS) before (incl.) and after (excl.) lesion masking in grey (GM) and white matter (WM) general structures.

Metric	GM			WM		
	HC	MS (incl.)	MS (excl.)	HC	MS (incl.)	MS (excl.)
FA	0.1301 ± 0.0069	0.1329 ± 0.0061 (*p* < 0.001)	0.1318 ± 0.0061 (*p* < 0.05)	0.286 ± 0.015	0.271 ± 0.026 (*p* < 0.001)	0.270 ± 0.026 (*p* < 0.001)
MD	0.787 ± 0.021	0.821 ± 0.037 (*p* < 0.001)	0.820 ± 0.037 (*p* < 0.001)	0.682 ± 0.014	0.713 ± 0.031 (*p* < 0.001)	0.712 ± 0.030 (*p* < 0.001)
FD	2.600 ± 0.014	2.607 ± 0.015 (*p* < 0.001)	2.603 ± 0.014 (*p* = 0.071)	2.528 ± 0.015	2.502 ± 0.029 (*p* < 0.001)	2.499 ± 0.032 (*p* < 0.001)

FDR-corrected *p*-values for statistically significant differences between MS and HC are shown below the metric. For FA in GM Welch, the t-test was used, while the Mann–Whitney U-test was used for the remaining comparisons.

In GM, FA was slightly higher in MS patients compared to HC, and the difference remained statistically significant both before (*p* < 0.001) and after lesion exclusion (*p* < 0.05). Conversely, WM showed a clear reduction in FA in MS for both conditions (*p* < 0.001), indicating disrupted microstructural organization. MD was significantly elevated in MS patients across both tissue types and masking conditions (all *p* < 0.001). FD was marginally higher in GM in MS before lesion masking (*p* < 0.001), but this difference became non-significant after lesion exclusion (*p* = 0.071). In WM, FD was consistently and significantly lower in MS patients before and after masking (both *p* < 0.001), suggesting reduced structural complexity beyond focal lesions.

Compared with sDTI (Supplementary Table S1), BSD produced consistently lower FA and MD values in both GM and WM across all groups. In GM, FA values in HC, MS (lesions included), and MS (lesions excluded): 0.1301 vs. 0.1338, 0.1329 vs. 0.1366, and 0.1318 vs. 0.1355; MD: 0.787 vs. 0.820, 0.821 vs. 0.854, 0.820 vs. 0.853·10^−3^ mm^2^/s for BSD vs. sDTI, respectively. In WM, FA values were 0.286 vs. 0.289, 0.271 vs. 0.274, 0.270 vs. 0.273, and MD were 0.682 vs. 0.698, 0.713 vs. 0.729, 0.712 vs. 0.728·10^−3^ mm^2^/s for BSD vs. sDTI in HC, MS-included and MS-excluded, respectively. Standard deviations were comparable across methods and groups, with BSD showing slightly smaller variability in some cases but no systematic shift.

### 3.2 T1-based FD in relation to DTI metrics

**Figure 3:**
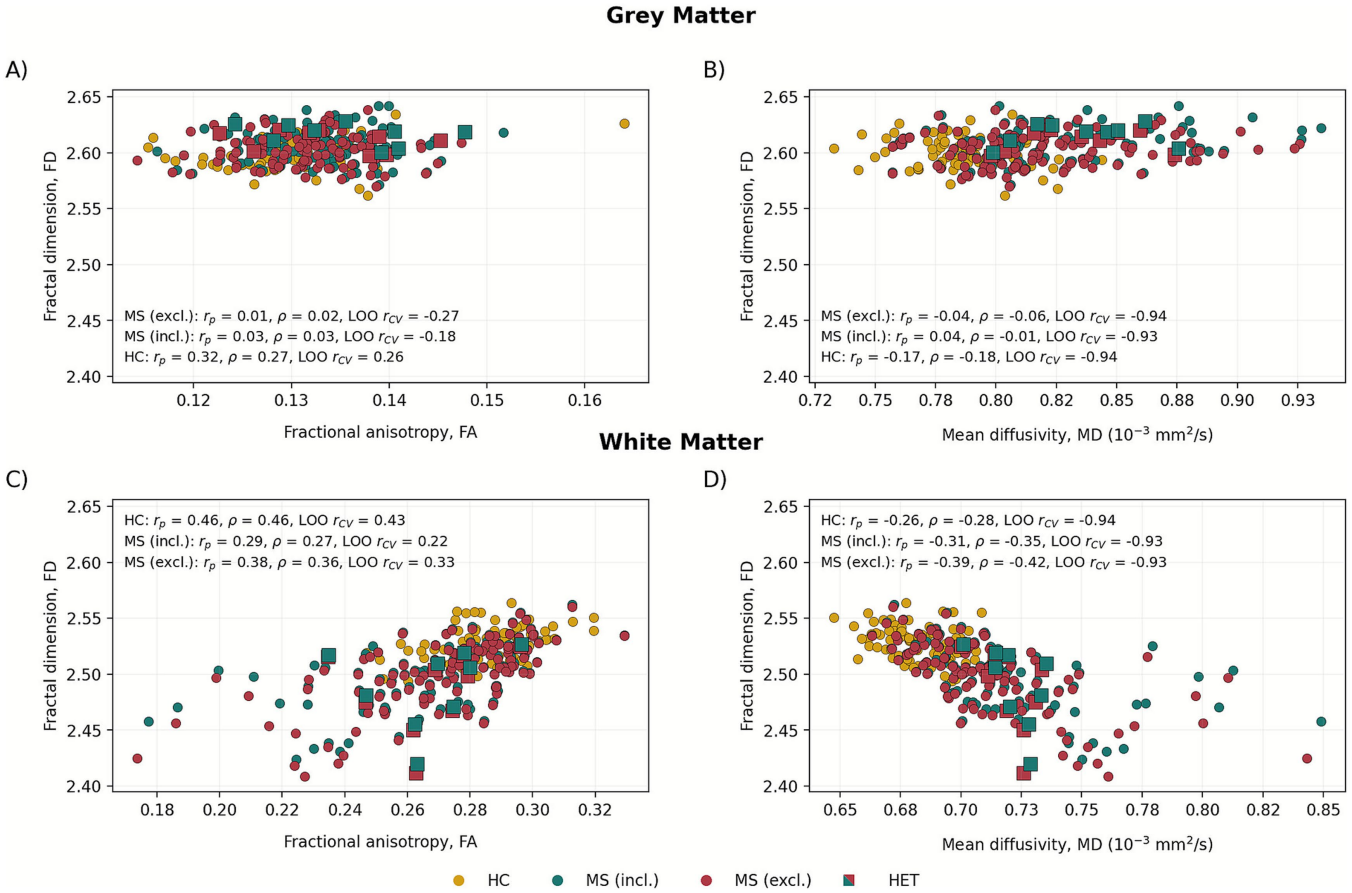
FD-DTI correlation plots in Grey **(A,B)** and white matter **(C,D)** in HC and MS cohort with division to lesions included (incl.) and excluded (masked out) (excl.) conditions. Patients treated with high-efficacy therapy (HET) are discerned as squares. On the plots, Pearson (*r_p_*), Spearman (*ρ*), and Pearson in leave-one-out cross-validation (*r_CV_*) correlation coefficients are shown.

Figure 3 shows FD in relation to DTI metrics in both GM and WM. Correlation metrics are shown in Table 3. In the HC group, FD showed moderate, significant, positive, robust, and stable correlations with FA in both GM and WM. FD-MD relationships were significant only in WM. They were consistent across Pearson, Spearman, and Huber analyses, while the effects (based on LOO-CV) were unstable. In Figure 3, HET and MET subjects were divided to visualize potential grouping. However, FD and DTI metrics were not significantly different (*p* > 0.05) between those two groups. Eventually, HET- and MET-treated patients were analyzed jointly.

**Table 3:** Partial correlation metrics of T1-based fractal dimension (FD) and BSD-DTI metrics of general structure (FA, MD) and White Matter skeleton with statistically significant probabilities of these correlations in the brackets.

Correlation metric	Group	GM				WM			
		FA	MD	FA (skel.)	MD (skel.)	FA	MD	FA (skel.)	MD (skel.)
Pearson, *r_p_*	HC	**0.32 (*p* < 0.05)**	−0.17	−0.034	0.097	**0.46 (*p* < 0.001)**	**−0.26 (*p* < 0.05)**	0.37	−0.14
Spearman, *ρ*	HC	**0.27 (*p* < 0.05)**	−0.18	0.24	−0.039	**0.46 (*p* < 0.001)**	**−0.28 (*p* < 0.001)**	0.41	−0.13
Huber slope	HC	0.78	−5.4E-6	0.27	3.4E-6	0.52	−9.9E-6	0.46	−7.1E-6
LOO, *r_CV_*	HC	0.26	−0.94	−0.14	−0.94	0.43	−0.94	0.31	−0.95
Pearson, *r_p_*	MS (incl.)	0.032	0.042	**−0.22** **(*p* < 0.05)**	**0.32** **(*p* < 0.001)**	**0.29 (*p* < 0.05)**	**−0.31 (*p* < 0.05)**	**0.45** **(*p* < 0.001)**	**−0.30** **(*p* < 0.05)**
Spearman, *ρ*	MS (incl.)	0.030	−0.0057	−0.14	**0.27** **(*p* < 0.05)**	**0.27 (*p* < 0.05)**	**−0.35 (*p* < 0.001)**	**0.43** **(*p* < 0.001)**	**−0.38** **(*p* < 0.001)**
Huber slope	MS (incl.)	0.15	3E-6	−0.28	3.9E-5	0.33	−3.2E-5	0.44	-6E-6
LOO, *r_CV_*	MS (incl.)	−0.18	−0.93	0.12	−0.68	0.44	−0.93	0.43	−0.90
Pearson, *r_p_*	MS (excl.)	0.0078	−0.043	−0.080	0.18	**0.36 (*p* < 0.001)**	**−0.39 (*p* < 0.001)**	**0.53** **(*p* < 0.001)**	**−0.40** **(*p* < 0.001)**
Spearman, *ρ*	MS (excl.)	0.021	−0.056	−0.021	0.13	**0.36 (*p* < 0.001)**	**−0.42 (*p* < 0.001)**	0.51	−0.43
Huber slope	MS (excl.)	0.12	−2.9E-6	−0.075	2E−5	0.49	-5.3E-5	0.46	−8.6E-6
LOO, *r_CV_*	MS (excl.)	−0.27	−0.94	−0.29	−0.91	0.33	−0.93	0.51	−0.91

As covariates, age and brain parenchymal fraction (BPF) were used (statistically different between HC and MS). *r_p,_* Pearson correlations coefficient; *ρ*, Spearman correlation coefficient; LOO *r_CV_*, Pearson coefficient obtained between observed and predicted values in leave-one-out cross-validation; GM, Grey Matter; WM, White Matter; FA, fractional anisotropy of a general structure; MD, mean diffusivity of a general structure; FA (skel.), fractional anisotropy of a WM skeleton; MD, mean diffusivity of a WM skeleton; HC, healthy controls; MS, multiple sclerosis patients; (incl.), lesions unmasked; (excl.), lesions masked out. Bold values highlight statistically significant differences between HC and MS with *p*-values in the brackets.

In HC, FD-FA correlations were moderate in both WM (*r_p_* = 0.46, *p* < 0.001) and GM (*r_p_* = 0.32, *p* < 0.05). MD showed a negative correlation with FD in WM (*r_p_* = −0.26, *p* < 0.05). These associations were largely consistent across Pearson and Spearman coefficients, suggesting linear and monotonic relationships. In MS patients, FA-based correlations became slightly weaker, particularly in WM, with significant positive correlations observed both before (*r_p_* = 0.29, *p* < 0.05) and after lesion masking (*r_p_* = 0.36, *p* < 0.001). Conversely, MD consistently showed significant negative correlations with FD in MS WM (*r_p_* = −0.31, *p* < 0.05), suggesting that higher tissue diffusivity corresponds to reduced structural complexity. Notably, lesion masking increased the strength of these associations in WM, highlighting the relevance of microstructural alterations beyond focal lesions. In GM, correlations in MS were weak and mostly non-significant, indicating that FD may be more sensitive to WM tissue integrity. The differences in correlation strength between HC and MS were tested in the Fisher Z test. They were found in GM for FD-FA (*p* < 0.05), but did not survive FDR correction.

Application of BSD resulted in a selective reconfiguration of the correlations between general structure DTI metrics and FD in comparison to sDTI (Supplementary Table S3). One association observed in sDTI was no longer significant after BSD (MS (lesions included) GM MD- GM FD (sDTI *r_p_* = 0.17, *p* < 0.05)). Conversely, one correlation absent in sDTI was revealed after BSD, such as GM FD-GM MD in HC, or remained similar regardless of the method, but was more robust after BSD.

### 3.3 FD residual redundancy analysis

To evaluate whether FD retains disease-related information beyond what is already explained by FA, MD, age, and BPF, we examined the distribution of adjusted FD residuals (FD ~ FA + MD + age + BPF) across HC and MS groups. If the covariates fully capture the disease-related variance, the residuals should be statistically indistinguishable between groups, indicating redundancy of group information. Conversely, persistent group separation in the residual space would imply that FD contains unique, non-redundant variance associated with MS pathology.

**Figure 4:**
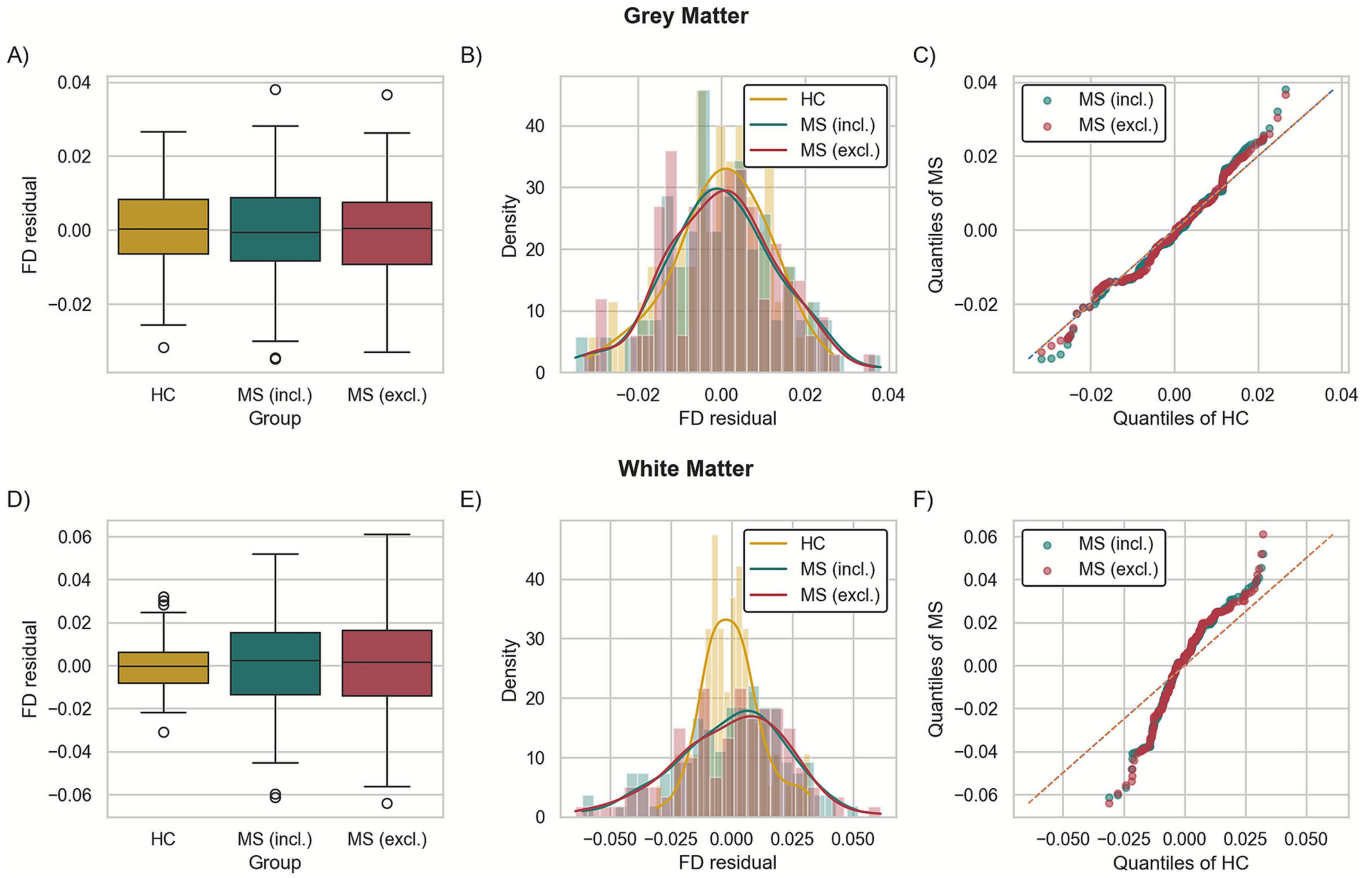
Fractal dimension (FD) residual analysis for the applied linear model (Equation 2): boxplots of residuals distribution **(A,D)**, histogram with KDE **(B,E)**, two-sample QQ-plots **(C,F)** in Grey **(A–C)** and white matter **(D–F)** for healthy controls (HC) and multiple sclerosis patients (MS) for unmasked data (incl.) and after lesion masking (excl.).

Residual analysis did not deliver statistically significant differences between HC and MS. However, a comparison of the FD residual distributions (Figures 4A,B,D,E) shows that their distributions differ. Two-sample QQ-plots analysis (Figures 4C,F) showed deviation from the y = x line, suggesting differences in residual distributions. Bootstrap analysis of means and medians (HC vs. MS) did not reveal significant differences, whereas statistically significant differences were found for the whole distributions in WM (*p* < 0.05). However, only comparison of HC and MS without lesion masking (incl.) survived FDR correction at a significance level of 0.05.

### 3.4 Classification performance based on general structure metrics

**Figure 5:**
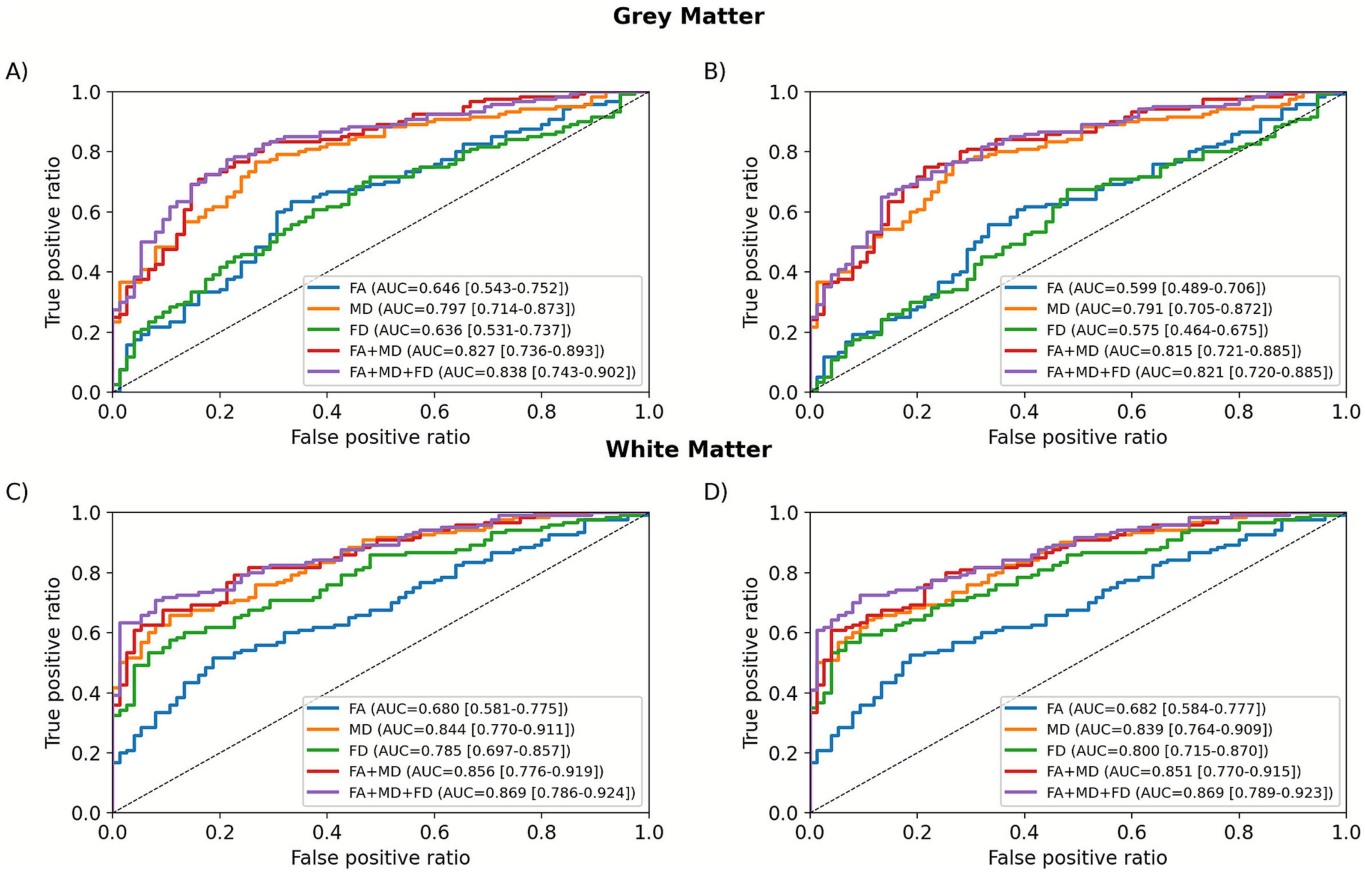
Receiver operating characteristic (ROC) curves for different combinations of general structure metrics in grey **(A,B)** and white matter **(C,D)** for data without **(A,C)** and with **(B,D)** lesion masking.

All T1-based and DTI metrics (FA, MD, FD) and their combinations were tested in ROC analysis. In this approach, metrics represent the general structures of WM and GM, and results are shown in Figure 5 and Table 4 (for sDTI ROC see Supplementary Figure S3). Among individual metrics, MD demonstrated the highest diagnostic performance in both GM (AUC = 0.797 for lesions included, 0.791 for lesions excluded) and WM (AUC = 0.844 for lesions included; 0.839 for lesions excluded), outperforming FA and FD. However, combining metrics led to a clear improvement in classification metrics. Specifically, the FA + MD + FD set achieved higher AUCs than any single metric, highlighting the added value of FD.

**Table 4:** Receiver operating characteristic (ROC) analysis results.

Tissue	Lesion status	Model	AUC	CI low	CI high	Youden	Sensitivity	Specificity	Accuracy
GM	incl.	FA	0.646	0.543	0.752	0.614	0.633	0.667	0.646
	incl.	MD	0.797	0.714	0.873	0.556	0.767	0.733	0.754
	incl.	FD	0.636	0.531	0.737	0.566	0.717	0.520	0.641
	incl.	FA + MD	0.827	0.736	0.893	0.622	0.725	0.827	0.764
	incl.	FA + MD + FD	0.838	0.743	0.902	0.558	0.775	0.787	0.779
	excl.	FA	0.599	0.489	0.706	0.619	0.558	0.667	0.600
	excl.	MD	0.791	0.705	0.872	0.560	0.767	0.733	0.754
	excl.	FD	0.575	0.464	0.675	0.594	0.675	0.520	0.615
	excl.	FA + MD	0.815	0.721	0.885	0.603	0.750	0.787	0.764
	excl.	FA + MD + FD	0.821	0.720	0.885	0.657	0.650	0.867	0.733
WM	incl.	FA	0.680	0.581	0.775	0.646	0.517	0.813	0.631
	incl.	MD	0.844	0.770	0.911	0.692	0.658	0.893	0.749
	incl.	FD	0.785	0.697	0.857	0.699	0.575	0.893	0.697
	incl.	FA + MD	0.856	0.776	0.919	0.703	0.675	0.907	0.764
	incl.	FA + MD + FD	0.869	0.786	0.924	0.669	0.708	0.920	0.790
	excl.	FA	0.682	0.584	0.777	0.644	0.525	0.813	0.636
	excl.	MD	0.839	0.764	0.909	0.697	0.642	0.893	0.738
	excl.	FD	0.800	0.715	0.870	0.716	0.567	0.933	0.708
	excl.	FA + MD	0.851	0.770	0.915	0.739	0.608	0.960	0.744
	excl.	FA + MD + FD	0.869	0.789	0.923	0.665	0.725	0.907	0.795
WM skeleton	NA	FA (skel.)	0.754	0.665	0.839	0.649	0.550	0.840	0.662
	NA	MD (skel.)	0.873	0.809	0.935	0.643	0.742	0.880	0.795
	incl.	FD (incl.)	0.785	0.697	0.857	0.699	0.575	0.893	0.697
	excl.	FD (excl.)	0.800	0.715	0.870	0.716	0.567	0.933	0.708
	NA	FA (skel.) + MD (skel.)	0.888	0.807	0.943	0.680	0.717	0.933	0.800
	incl.	FA (skel.) + MD (skel.) + FD (incl.)	0.900	0.822	0.943	0.641	0.758	0.973	0.841
	excl.	FA (skel.) + MD (skel.) + FD (excl.)	0.903	0.826	0.945	0.625	0.775	0.973	0.851

GM/WM, grey/white matter; incl., metrics obtained without lesion masking; excl., metrics obtained with lesion masking; AUC, area under curve; CI, 95% confidence interval; Youden, Youden index (discriminating threshold).

The best overall performance was obtained using the FA + MD + FD model in WM under the “lesions excluded” condition, achieving an AUC of 0.8689 (CI: 0.7888–0.9231), sensitivity of 0.725, specificity of 0.9067, and accuracy of 0.7949. The same model in the “lesions included” condition reached a similar AUC (0.8686), although with slightly higher specificity (0.92 vs. 0.9067) and accuracy (0.7897). For GM, performance was moderate, with the best results also seen for the FA + MD + FD model under the “lesions included” condition (AUC = 0.8380, CI: 0.7429–0.9023). However, the accuracy and specificity remained lower than those for WM, suggesting weaker separation of MS-related changes in GM diffusion metrics.

Sensitivity-specificity balance was further assessed using the Youden Index. The highest Youden values were observed for WM models, especially for FA + MD + FD (lesions excluded: Youden Index = 0.6646), confirming its good discriminative balance. Some FD-based models showed high specificity (up to 0.933), but at the expense of sensitivity, indicating a strong bias toward correctly classifying healthy controls.

Compared with sDTI, BSD produced the strongest improvements in ROC metrics in GM, particularly for FA, which showed the largest relative increases in AUC, sensitivity, and specificity (+1.94%/+1.73%, −3.95%/+13.43%, and +8%/−8% for lesions included/excluded, respectively). MD also improved consistently in GM, with AUC changes of +0.95%/+0.65%, sensitivity +5.43%/+5.43%, and accuracy +4.08%/+4.08% for lesions included/excluded, respectively. Combined metrics were similarly enhanced: FA + MD increased by +0.90%/+0.79% in AUC, while FA + MD + FD changed by +1.76%/+1.39% for lesions included/excluded, respectively. In WM, improvements were most robust for MD and FA + MD. MD showed AUC gains of +0.91%/+0.94% and the largest increases in specificity: +11.94%/+11.94% and accuracy: +0.68%/+0.69%, while FA + MD improved by +0.95%/+1.10% in AUC, with specificity changes of +8.82%/+8.33% and accuracy +1.34%/+1.38% for lesions included/excluded, respectively. Conversely, FA in WM exhibited small negative changes (AUC −0.16%/−0.08%, accuracy −0.81%/0% for lesions included/excluded, respectively), and models including FD tended to show slight declines in both conditions. Overall, BSD enhanced MD-based and FA + MD metrics across tissue types, improved FA-derived metrics in GM, and produced minimal or negative effects on FA and FD-augmented models in WM compared to sDTI.

### 3.5 TBSS-based fractal dimension

To determine group WM core FD, TBSS analysis was conducted separately for MS and HC. Group mean FA maps were skeletonized (Supplementary Figure S1), and FD was calculated based on the skeletons. The skeletonization FA threshold of 0.2 was chosen following the standard TBSS procedure described by Supplementary Figure S4 (22). To verify the robustness of our results, we repeated the skeletonization using thresholds of 0.15 and 0.25. The mean FA skeleton masks showed Dice overlaps of 0.93 (0.15 vs. 0.20) and 0.94 (0.20 vs. 0.25), with 100% spatial overlap of the 0.20 mask within the 0.15 skeleton. Based on the dendrogram, localizations of voxels are stable for thresholds 0.15 and 0.20, while 0.25 restricts the skeleton’s spread (Supplementary Figure S4). Thus, the skeleton structure, the spatial pattern of significant clusters, and HC vs. MS difference patterns remained qualitatively consistent and confirmed 0.2 as a reasonable threshold.

**Figure 6:**
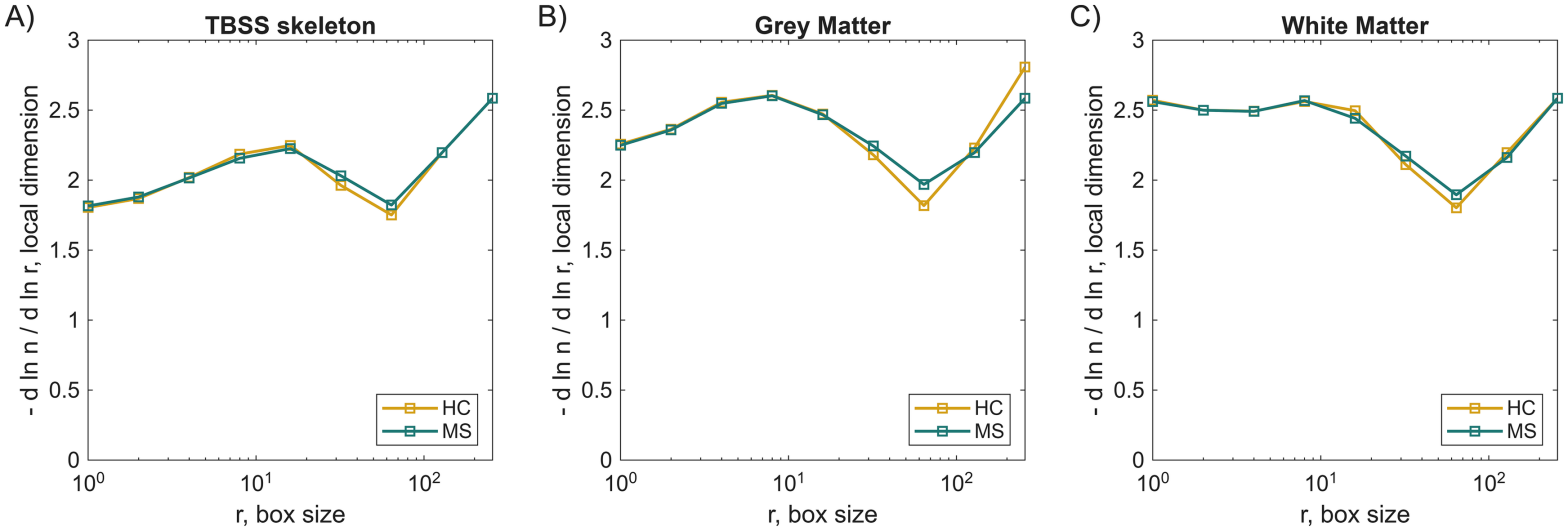
Local dimension (−d ln(n)/d ln(r)) as a function of a box size in the boxcounting method used to determine fractal dimension in group TBSS skeletons **(A)**, and T1-based Grey **(B)** and white matter **(C)** obtained from example individuals.

Figure 6 shows the ratio of derivatives of the number of boxes needed to cover the skeleton, *n*, and box size, *r*, as a function of 1/*r*. For comparison, the same dependency was shown in Figure 6 for T1-based WM and GM masks. It can be seen that skeletonized WM does not exhibit a linear component, as in the case of T1-based analysis. It means that FD is not scale-invariant, which is actually common in biological structures such as the brain. WM skeleton is not a “true fractal” but exhibits multifractal behavior. This suggests that we observe different structural properties at different spatial scales. Therefore, FD was calculated from relation *n*(*r*) within four ranges (limited by the image size according to the *boxcount* function in MATLAB): 1–2; 1–4; 1–8; 8–16. All results, including DTI metrics, are shown in Table 5. It can be seen that for very small box sizes, FD was slightly higher in MS. Increasing r leads to equal MS and HC FDs for medium box size and higher FD in HC cohort for the most linear part of Figure 6A (range 8–16). This means that for smaller local dimensions, the WM core in MS is more complex or simply rough. As the local dimension increases, fewer local details can be captured, and in this case, the global morphology becomes more complex in HC. This can also suggest a more coherent topology.

**Table 5:** Group mean fractional anisotropy (FA), mean diffusivity (MD), and fractal dimension (FD) values in the whole skeleton and in significant voxels obtained for different models in healthy controls (HC) and multiple sclerosis (MS) cohort.

Metric	Model	n_voxels_	HC	MS	Cohen’s d
FD (whole skeleton)	box size: 1–2	–	1.805	1.815	–
	box size: 1–4	–	1.868	1.879	–
	box size: 1–8	–	1.946	1.948	–
	box size: 8–16	–	2.266	2.224	–
FA (whole skeleton)	MS vs. HC	81,253	0.409 ± 0.023	0.386 ± 0.030	−0.84
MD (whole skeleton) (10^−3^ mm^2^/s)	MS vs. HC	81,253	0.666 ± 0.020	0.712 ± 0.045	1.23
FA (significant voxels)	MS vs. HC	53,111	0.420 ± 0.023	0.392 ± 0.034	−0.99
	MS vs. HC + Age	50,169	0.422 ± 0.023	0.393 ± 0.034	−1.01
	MS vs. HC + Age + SDMT	55,912	0.418 ± 0.023	0.389 ± 0.034	−0.98
	MS vs. HC + Age + SDMT + EDSS	42,589	0.433 ± 0.023	0.400 ± 0.036	−1.02
MD (significant voxels) (10^−3^ mm^2^/s)	MS vs. HC	53,111	0.679 ± 0.020	0.733 ± 0.052	1.30
	MS vs. HC + Age	50,169	0.680 ± 0.020	0.736 ± 0.053	1.30
	MS vs. HC + Age + SDMT	55,912	0.677 ± 0.020	0.731 ± 0.051	1.29
	MS vs. HC + Age + SDMT + EDSS	42,589	0.682 ± 0.021	0.741 ± 0.055	1.31

Covariates used in a given model are mentioned after the plus sign. All FA and MD values are significantly different between HC and MS (*p* < 0.001). Effect size (Cohen’s d) is also presented for the difference between HC and MS.

BSD and sDTI produced highly concordant HC-MS differences in FD, FA, and MD across both whole-skeleton and significant-voxel analyses (Supplementary Table S4). BSD introduced small systematic downward shifts in FA and MD but did not meaningfully alter effect sizes or spatial patterns of group differences.

### 3.6 Modeling the variability of white matter core FA

**Figure 7:**
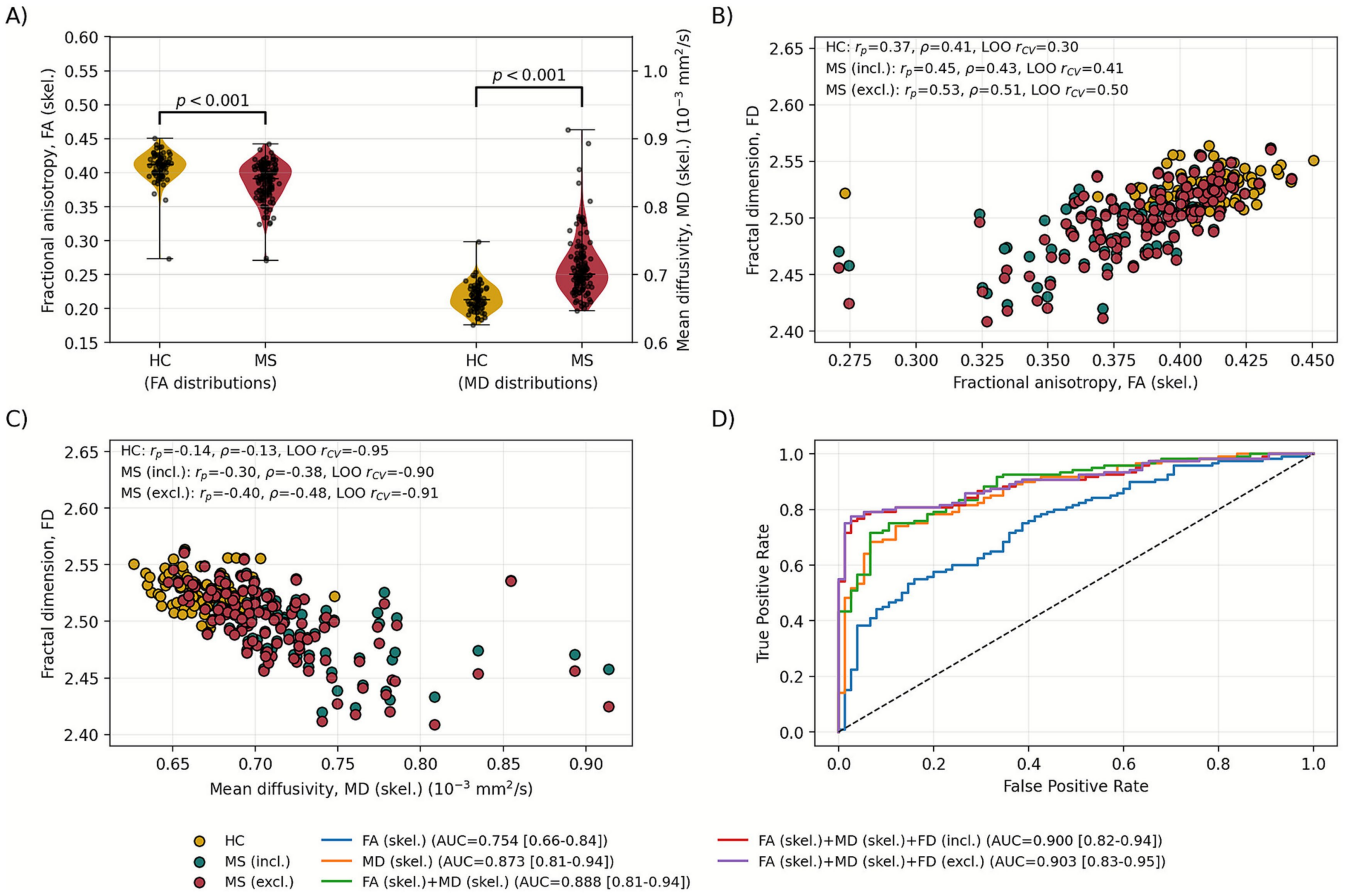
White matter core fractional anisotropy (FA (skel.)) and mean diffusivity (MD (skel.)) distributions **(A)**, their correlations with T1-based fractal dimension (FD) **(B,C)**, and receiver operating characteristic (ROC) curves for them and their combinations **(D)**. On the ROC panel area **(D)**, the under-curve (AUC) and its 95% confidence interval are shown. *p*-values were obtained in the Mann–Whitney U test.

Average FA and MD values in a common mean skeleton mask (in this approach, MS and HC subjects had one group mean skeleton) were calculated (Figure 7A; Table 5). Statistically significant differences between MS and HC were found for both FA and MD.

A statistically significant relationship between DTI metrics and clinical variables- EDSS score, SDMT score, and age, within the mean WM skeleton of a group consisting of both MS and HC, was investigated. Firstly, the correlation among covariates was examined (Supplementary Figure S2), and the variance inflation factor (VIF) was calculated (Supplementary Table S2). In MS, EDSS and SDMT are slightly negatively correlated (*r_p_* = −0.107), not significantly (*p* = 0.232), suggesting that cognitive performance is not strongly related to disability in this sample. EDSS versus age showed a weak positive correlation (*r_p_* = 0.148), not significant (*p* = 0.098), which indicates that older MS patients tend to have slightly higher disability scores. Finally, SDMT and age exhibited moderate negative correlations (*r_p_* = −0.361 and *r_p_* = −0.175 for MS and HC, respectively), significant for MS (*p* < 0.001) and not significant for HC (*p* = 0.13). However, results show that aging in MS additionally increases cognitive performance. All VIFs were ~1, which means that covariates (EDSS, SDMT, Age) are not collinear and can be safely included together in the same model.

Based on Table 5, it can be seen that the model with all covariates is the most restrictive in terms of the number of significantly different voxels between HC and MS. For this model, the largest MD and FA values were observed, which means that covariate-controlled differences occur in more anisotropic regions, where the largest differences between the two cohorts were obtained. Most voxels are significant in all four models (41,161 voxels), but some voxels are distinguished for a single model (see Supplementary Figure S5). Very large effect sizes calculated based on Cohen’s d coefficient are visible between HC and MS. Larger values were observed for MD in both whole skeleton and significant voxels, whereas the overall largest value occurred for MD when controlling for all covariates.

### 3.7 Combining white matter core metrics with general structure FD

Fractal dimension of WM general structure (T1-based) was correlated with DTI metrics obtained in TBSS skeleton (Figures 7B,C; Table 3). In the case of WM core MD, partial correlation coefficients were very similar to those obtained for general structure in MS, while reduced for HC. For WM core FA, the HC group showed decreased correlation with WM general structure FD. However, in the MS group, correlation between T1-based FD and WM core FA was visibly stronger than for FD-FA from general structure (*r_p_* = 0.45 vs. *r_p_* = 0.29 for lesions included and *r_p_* = 0.53 vs. *r_p_* = 0.38 for lesions excluded). All correlations were robust and stable (Table 3). Furthermore, WM skeleton-based FA and MD were correlated with T1-based FD of GM. No associations were found in the HC group and MS with excluded lesions, neither for FA (skeleton) nor MD (skeleton). However, in the case of MS with lesions within the mask, significant, negative, robust, but unstable correlations were found for both FA (skeleton) and MD (skeleton) (FA *r_p_* = −0.22 and MD *r_p_* = 0.32).

Within the WM interior region, the classification model performance improved compared to whole-brain analysis (Figure 7D; Table 4). Among individual metrics, skeleton MD exhibited the strongest predictive capability, achieving an AUC of 0.873 (CI: 0.809–0.935), sensitivity of 0.742, specificity of 0.880, and an overall accuracy of 0.795. This confirms that MD remains the most sensitive single biomarker for detecting MS-related microstructural changes in deep WM regions.

Skeleton FA demonstrated moderate discriminative power (AUC = 0.754), with good specificity (0.840) but lower sensitivity (0.550), indicating its tendency to better identify healthy controls than MS cases. The combination of features led to substantial improvements in classification performance. The skeleton-based FA + MD model achieved an AUC of 0.888 (CI: 0.807–0.943) and a markedly high specificity of 0.933, indicating strong discriminative capacity even without FD. However, the best results were achieved using FA (skeleton) + MD (skeleton) + FD (general structure), both with and without lesions. The top-performing model (FA + MD + FD with excluded lesions) reached an AUC of 0.903 (CI: 0.826–0.945), specificity of 0.973, sensitivity of 0.775, and the highest overall accuracy (0.851). This indicates that multimodal diffusion features provide complementary information and considerably enhance both sensitivity and specificity.

Analyses based on skeletonized diffusion metrics revealed several differences between sDTI and BSD. Several associations that were not detectable in sDTI became significant after BSD: (1) HC WM: FA(skeleton)-FD (*r_p_* = 0.37, *p* < 0.05); (2) MS (incl.) GM: FA(skeleton)-FD (*r_p_* = −0.22, *p* < 0.05) and MD(skeleton)-FD (*r_p_* = 0.32, *p* < 0.001); (3) MS (excl.) GM: MD(skeleton)-FD (*r_p_* = 0.18, *p* < 0.05); (4) MS (excl.) WM: FA(skeleton)-FD (*r_p_* = 0.53, *p* < 0.001).

## 4 Discussion

This study investigated the feasibility and diagnostic relevance of T1-based FD in conjunction with conventional DTI metrics (FA and MD) for describing MS-related microstructural alterations in both general brain tissue structures and deep WM skeletons. In GM, MS patients showed higher FD, MD, and FA, which may indicate functional compensation mechanisms via microstructural reorganization. In WM, MS showed lower FA and FD and higher MD, suggesting demyelination and inflammation. FD demonstrated distinct and complementary sensitivity to structural abnormalities, particularly in WM, and its integration with DTI-derived features considerably improved classification performance. The combination of FA, MD, and FD achieved the highest diagnostic accuracy, particularly in the WM skeleton-based approach, indicating that multimodal geometric-microstructural assessment may provide a more comprehensive characterization of MS pathology.

### 4.1 Methodology choice

In our previous studies (25, 26), it was shown that BSD correction can be crucial for local microstructure analysis in smaller ROIs and may enhance the distinction between MS and HC. This is further supported (27), who demonstrated that the classical implementation of BSD significantly altered FA/MD values, bringing them closer to reference measurements, and hence developed a deep learning-based version for more convenient BSD applications. In the present study, we build on this evidence for the BSD application in MS, showing results, while corresponding sDTI results are available in the Supplementary materials. Across diffusion metrics, BSD yielded lower mean FA and lower mean MD in both GM and WM and both HC and MS, indicating that conventional sDTI in this dataset tends to overestimate both anisotropy and diffusivity. When the effective *b*-value is lower than the nominal, which is common in peripheral brain regions, sDTI yields artificially elevated MD. FA decreased under BSD due to the method’s ability to correct anisotropic *b*-matrix distortions, including direction-dependent gradient magnitude errors and off-diagonal terms, thereby altering the eigenvalue structure of the diffusion tensor rather than uniformly scaling it. These reductions were most pronounced in GM, likely reflecting the greater numerical sensitivity of low-anisotropy and highly orientation-dispersed tissue to direction-dependent gradient errors and eigenvalues sorting. Moreover, GM is located more off-centrally with respect to the magnet’s isocenter, where magnetic field gradients are more non-uniform. Second, BSD both reveals biologically meaningful associations and removes spurious ones. Newly appearing correlations in MS involve FA (skeleton) and MD (skeleton) metrics, which under sDTI were essentially absent or highly unstable (Supplementary Table S3). Intriguingly, BSD also strengthened the cross-tissue coupling between skeleton WM, MD, and GM FD, indicating that GM morphological complexity is more tightly linked to the WM core diffusivity than sDTI implied. This cross-region dependence becomes detectable only when systematic errors are removed and may reflect shared degeneration pathways or long-range structure–function coupling in MS. Finally, BSD improves patient/control classification performance, with the strongest and most consistent gains in GM and for MD-based or combined diffusion metrics, while effects in WM are metric-dependent and generally smaller.

In the study, lesions were deliberately not masked, as the goal was to capture the global structural complexity of the brain, including both normal-appearing tissue and lesioned areas. This makes FD more reflective of the overall disease burden. To assess the stability of results, we compared them with those coming from images with masked-out lesions. Given the relatively low lesion load in our cohort (mean LPF 0.83%), their contribution to global average FD metrics was minimal. Discriminative sensitivity is preserved or improved after lesion masking, depending on the tissue. This is supported by previous work showing that cortical FD remains predictive of clinical outcomes even after adjusting for T2 lesion volume (10). This topic will be discussed in the next section in relation to the results shown in this study.

Lastly, our study focused on whole-brain measures without regional analysis. Whole-brain measures, including FD, have been successfully applied in previous MS studies (13) and captured the cumulative impact of local alterations. Previous work has suggested that regional FD may provide higher sensitivity to subtle cortical pathology (10). Global measures offer robustness against local segmentation errors, are more reproducible, allow direct comparison with whole-brain DTI metrics such as FA and MD, and can serve as fast, preliminary classifiers. Future studies could extend these analyses to regional FD to investigate more localized microstructural changes in MS. Regional cortical FD analysis represents an important next step, as it may provide finer-grained information on localized microstructural changes, similar to how region-specific measures of cortical thickness or diffusion metrics have been used in MS and other neurodegenerative diseases to detect subtle pathology (e.g., cortical thinning in Alzheimer’s disease or regional FA reductions in MS). Integrating both global and regional approaches could thus offer a more comprehensive assessment of disease burden.

### 4.2 Impact of lesion masking on the integrity-complexity relationship

A common approach in MRI studies of MS is to mask or fill lesions before analysis to obtain “pure” WM or GM measures. While useful for standardization in, for example, volumetric assessments of GM and WM, this practice imposes an artificial separation between normal-appearing and diseased tissue, even though lesions and NAWM interact and jointly contribute to global brain alterations. This can be confirmed by the moderate correlation between WM skeleton FA and GM FD.

In our study, average FD and DTI values and their correlations were minimally affected by lesion masking, but the significant redistribution of correlations after masking suggests that lesions carry distinct microstructural variance that strongly modulates FD-DTI relationships. In WM, lesion masking increases correlations with general structure and skeleton metrics, but diminishes skeleton-based correlations in GM. This suggests that lesions contribute meaningfully to the complexity signal, and that whole-brain FD may serve as a complementary marker of global structural disruption. Retaining lesions in the analysis does not necessarily reduce interpretability but can capture the integrated nature of MS pathology.

The discriminative ability of FD appeared to depend on whether lesions were masked out or not (Figures 5, 7). In WM, FD performed worse under the “lesions included” condition. In GM, FD demonstrated higher performance, but overall performance in GM was worse than in WM. The best discrimination between MS and HC was obtained with skeleton-based metrics, where the difference between “lesions included” and “lesions excluded” conditions was minimal, but the best accuracy was achieved for the latter. When the correlation between WM skeleton FA and WM FD is additionally analyzed, it can be concluded that greater integrity of the WM core increases WM general structure complexity. Negative correlation between WM skeleton FA/MD and GM FD, which had opposite sign to what was observed in WM, indicates that higher integrity and diffusivity of WM core is connected with lower FD of GM calculated for “lesions included” condition. Since this association did not persist after excluding lesions, it can be concluded that WM core condition and lesion load are related.

### 4.3 FD of the white matter interior (skeleton)

T1-based FD was higher in GM than in WM, indicating greater structural intricacy in cortical regions. This result is consistent with neuroanatomical complexity and aligns with previous findings that attribute higher FD in GM to its convoluted gyri and sulci (1). Applying FD to TBSS skeletons entails certain interpretational challenges, since skeletonization inevitably suppresses part of the geometric variability that FD is designed to capture. Nevertheless, we considered this approach valuable because several studies have shown that skeleton-derived FD provides unique insights into WM organization (28, 29) demonstrated its sensitivity to age, hemisphere asymmetry, and sex beyond volumetric effects, while (30) showed that skeleton FD can detect microstructural alterations in traumatic brain injury when general structure (global) or surface FD and DTI metrics do not. Likewise, (7) confirmed that cerebellar skeleton FD reflects branching complexity linked to morphological features such as branch and node counts. Even studies that did not directly compute FD on the WM skeleton, such as (12), explicitly suggested that skeleton-based FD may represent a promising avenue by reducing the variance introduced by surrounding tissue.

TBSS-based analysis conducted separately for both groups revealed that the WM skeleton does not exhibit a constant FD across scales, indicating multifractal behavior. This observation reflects the complex, scale-dependent nature of WM structure, especially in diseased tissue. This may be particularly relevant in MS, where focal lesions and diffuse pathology create heterogeneous spatial patterns. FD of the whole skeleton increased progressively with larger box-size ranges, reflecting expected scaling properties of the fractal measurement, and was higher for HC for larger scales. Conversely, FA and MD extracted from skeleton voxels showed stronger group separation, with large effect sizes (FA: -1.02, MD: 1.31). Combined with the general structure of FD, they can be valuable diagnostic measures.

### 4.4 Exploratory associations of FA with clinical variables in WM interior

To evaluate the potential effects of clinical variables on FA, MS, and HC groups, they were combined and analyzed using TBSS. We hypothesized that higher EDSS scores would be associated with reduced FA in MS patients, reflecting increased structural damage corresponding to disease severity. We also tested the correlation between SDMT and FA in both HC and MS groups to investigate whether cognitive performance influenced FA and to assess WM involvement in cognition. The covariates’ associations are very widespread, almost global across the WM skeleton (Supplementary Figure S5). In TBSS, the skeleton represents the core of major WM tracts, so a high percentage of significant voxels indicates associations between clinical variables with FA in almost all tracts. This could mean that the disease effects in MS are not localized but instead diffusely affect the white matter. Effects of SDMT, EDSS, and age are partially visible in the same voxels. This suggests that these covariates are associated with FA changes in overlapping brain regions. For example, voxels where EDSS is negatively correlated with FA might also be correlated with SDMT or age. There is a shared neurobiological substrate where disease severity (EDSS), cognitive performance (SDMT), and aging converge. It implies that these factors are not independent. Older patients may perform worse on the SDMT and have higher EDSS. WM degradation may reflect a combined burden of aging, disease progression, and cognitive impact.

Building on this, highly significant correlations of FA (skeleton) and FD (both lesions included and excluded conditions), controlling for BPF and age, that did not occur for HC, together with the high discriminative power of these metric combinations, may indicate that they can be sensitive to functional and cognitive decline in MS.

### 4.5 Comparison of the two approaches

Our results using global T1-based WM and GM masks revealed a different pattern of FD alterations in MS. In WM, FD was lower in MS than in HC, accompanied by a concurrent decrease in FA and increases in MD. This contrasts with GM, where FD, FA, and MD were higher in MS. An additional analysis directly comparing “lesions included” and “lesions excluded” conditions clarified the role of focal pathology. Importantly, lesion load in our cohort was low (~0.8% of parenchymal volume), reinforcing that global FD alterations cannot be explained simply by lesion burden but rather reflect broader changes in tissue microstructural complexity. This can be confirmed by an increased MS FA of GM, regardless of lesion-masking status.

The observed T1-based WM FD reduction in MS in comparison to HC likely reflects the inclusion of peripherally located voxels with spatially coherent microstructural disruptions at the WM/GM interface, which are particularly sensitive to demyelination, axonal loss, and microstructural simplification (31). Loss of fiber density and organization in these juxtacortical regions reduces structural complexity, thereby decreasing FD. In case of GM, juxtacortical and subcortical lesions, along with inflammatory processes and dendritic remodeling, can enhance microstructural irregularity in cortical regions, resulting in higher FD values in MS compared to HC (32). These divergent patterns between WM and GM underscore the value of global mask analysis, which captures the combined effects of diffuse and interface pathology reflected in GM measures and yields greater discriminative sensitivity compared to the masked-out lesions approach.

Conversely, TBSS analysis showed visible reductions in FA in MS without substantial FD changes. This supports the notion that WM interior damage primarily involves diffuse demyelination or axonal loss without introducing substantial geometric complexity change detectable by FD (33). In other words, the perivenous origin of lesion formation, followed by confluent and radial plaque formation, can be reflected in the intact WM core regularity (34). Furthermore, FA-FD correlations and their discriminative power increase after lesion masking. Thus, FD appears particularly sensitive to pathological alterations at tissue interfaces, where lesions are more likely to disrupt local structural heterogeneity, whereas central tracts may show decreased anisotropy without appreciable changes in fractal geometry. This is also consistent with the concept of “surface-in” gradient of neuronal and axonal loss in GM, and can be indicative of an early stage of the disease (32). Taken together, the combination of global T1-based masks and TBSS provides a more nuanced characterization of MS-related microstructural changes. This integrative approach can enhance the ability to detect subtle disease-related structural complexity changes that may not be apparent when using a single analytic framework, but more studies on the FD of the general WM/GM structure, FD of the WM/GM surface, and FD of the WM interior are needed to explore this topic more deeply (for example using different MRI modalities).

### 4.6 Limitations and possible future directions

This study has several limitations. First, we examined only global GM and WM fractal dimension, which is suitable for capturing widespread structural complexity but limits anatomical specificity and prevents identification of regional contributors to group differences. Second, the cross-sectional design precludes conclusions about longitudinal change or prognostic value. Third, the cohort consisted mainly of individuals with low disability, which limits generalizability and reduces the sensitivity to detect relationships between FD, DTI, and clinical measures.

Although FD-DTI correlations were central for assessing complementarity, these analyses cannot establish causal or mechanistic links between geometric complexity and diffusion properties. Lesion inclusion and exclusion each influence global metrics, and neither fully isolates diffuse pathology (lesion exclusion removes focal plaques, but the remaining NAWM/NAGM can include perilesional and subtle diffuse abnormalities, meaning that the resulting FD still reflects heterogeneous pathology rather than an isolated diffuse signal). Moreover, all analyses were derived from a single scanner and processing pipeline. Although mask type, lesion masking, and BSD correction were examined, external validation is required to confirm the independence of BSD-derived measures.

Interpretation from a clinical perspective is also constrained. Global FD captures integrated structural irregularity but does not indicate which tracts or cognitive domains it reflects, and low variability in clinical scores limits the detection of clinically meaningful associations. Exploratory links between FA, FD, and cognitive or functional measures; hence, they require replication in larger and more heterogeneous samples.

Future studies should incorporate regional and multi-scale FD analyses, apply longitudinal designs to test sensitivity to disease progression, and integrate FD with additional microstructural or connectomic measures. Multi-site harmonization, including but not limited to BSD-based approaches, will be essential for establishing the robustness of FD-DTI relationships in broader clinical and research contexts.

## Data Availability

The raw data supporting the conclusions of this article will be made available by the authors, without undue reservation.
