# Supplementary material for: T1- and diffusion tensor-based fractal dimension of white and grey matter in multiple sclerosis
Source: Front Neurol. 2026 Jan 7;16:1618319. doi: 10.3389/fneur.2025.1618319 (PMC12819621; doi:10.3389/fneur.2025.1618319)
Supplement: Supplementary file 1 [file Data_Sheet_1.pdf]

T1- and diffusion tensor-based fractal dimension of White and Grey Matter in Multiple Sclerosis

Weronika Mazur-Rosmus[1*], Zofia Schneider[1], Agnieszka Słowik[2], Artur T. Krzyżak[1*]

[1] LaTiS NMR Tomography and Spectroscopy Laboratory, Department of Fossil Fuels, Faculty of Geology, Geophysics and Environmental protection, AGH University of Krakow, Krakow, Poland
[2] UJ CM: Department of Neurology, Jagiellonian University Medical College, University Hospital in Krakow, Krakow, Poland

*** Correspondence:**
Corresponding Author
akrzyzak@agh.edu.pl

Supplementary Information

Table of contents

Table S1. Mean values of fractional anisotropy (FA) and mean diffusivity (MD) obtained for sDTI, fractal dimension (FD) analysis.

Table S2. Correlation matrix with *p* (in the brackets) and variance inflation factor (VIF) for three analyzed covariates.

Table S3. Partial correlation metrics of T1-based fractal dimension (FD) and sDTI metrics of general structure (FA, MD) and White Matter skeleton with statistically significant probabilities of these correlations in the brackets. As covariates, age and brain parenchymal fraction (BPF) were used (statistically different between HC and MS). r_p – Pearson correlations coefficient, ρ – Spearman correlation coefficient, LOO r_{CV}- Pearson coefficient obtained between observed and predicted values in leave-one-out cross-validation, GM- Grey Matter, WM- White Matter, FA- fractional anisotropy of a general structure, MD- mean diffusivity of a general structure, FA (skel.)- fractional anisotropy of a WM skeleton, MD- mean diffusivity of a WM skeleton, HC- healthy controls, MS- multiple sclerosis patients, (incl.)- lesions unmasked, (excl.)- lesions masked out.

Table S4. Group mean fractional anisotropy (FA), mean diffusivity (MD) and fractal dimension (FD) values in the whole skeleton and in significant voxels obtained for different models in healthy controls (HC) and multiple sclerosis (MS) cohort obtained for sDTI. Covariates used in a given model are mentioned after the plus sign. All FA and MD values are significantly different between HC and MS ($p<0.001$). Effect size (Cohen's d) is also presented for the difference between HC and MS.

Figure S1. Skeletons from TBSS analysis in FSL overlaid on MNI152_T1_1mm image from FSL library for MS patients (upper row) and HC group (bottom row). Skeletons were obtained from FA map from diffusion tensor calculated using sDTI (A and C) and BSD approach (B and D).

Figure S2. Correlation plots for covariates: Expanded Disability Status Scale (EDSS), Symbol Digit Modalities Test (SDMT), Age.

Figure S3. Receiver operating characteristic (ROC) curves obtained for sDTI approach.

Figure S4. Dendrogram showing thresholds similarity based on Dice coefficient across models. X-axis shows 1-Dice value (shortest linkage = more similar).

Figure S5. Example slices of significant voxels in FMRIB58 space obtained in a hierarchical model for fractional anisotropy (FA) variability.

Table S1. Mean values of fractional anisotropy (FA) and mean diffusivity (MD) obtained for sDTI, fractal dimension (FD) analysis.

Metric	GM			WM		
	HC	MS (incl.)	MS (excl.)	HC	MS (incl.)	MS (excl.)
FA	0.1338 ± 0.0072	0.1366 ± 0.0065 (p<0.05)	0.1355 ± 0.0065 (p<0.05)	0.289 ± 0.015	0.274 ± 0.026 (p<0.001)	0.273 ± 0.026 (p<0.001)
MD	0.820 ± 0.026	0.854 ± 0.035 (p<0.001)	0.853 ± 0.035 (p<0.001)	0.698 ± 0.015	0.729 ± 0.032 (p<0.001)	0.728 ± 0.031 (p<0.001)
FD	2.600 ± 0.014	2.607 ± 0.015 (p<0.001)	2.603 ± 0.014 (p<0.05)	2.528 ± 0.015	2.502 ± 0.029 (p<0.001)	2.499 ± 0.032 (p<0.001)

Table S2. Correlation matrix with *p* (in the brackets) and variance inflation factor (VIF) for three analyzed covariates.

MS				
	EDSS	SDMT	Age	VIF
EDSS	1.00 (1.00)	-0.107 (0.232)	0.148 (0.098)	1.03
SDMT	-0.107 (0.232)	1.00 (1.00)	-0.361 (<0.001)	1.15
Age	0.148 (0.098)	-0.361 (<0.001)	1.00 (1.00)	1.17
HC				
EDSS	--	--	--	--
SDMT	--	1	-0.175 (0.13)	1.03
Age	--	-0.175 (0.13)	1	1.03

Table S3. Partial correlation metrics of T1-based fractal dimension (FD) and sDTI metrics of general structure (FA, MD) and White Matter skeleton with statistically significant probabilities of these correlations in the brackets. As covariates, age and brain parenchymal fraction (BPF) were used (statistically different between HC and MS). r_p – Pearson correlations coefficient, ρ – Spearman correlation coefficient, LOO r_{CV}- Pearson coefficient obtained between observed and predicted values in leave-one-out cross-validation, GM- Grey Matter, WM- White Matter, FA- fractional anisotropy of a general structure, MD- mean diffusivity of a general structure, FA (skel.)- fractional anisotropy of a WM skeleton, MD- mean diffusivity of a WM skeleton, HC- healthy controls, MS- multiple sclerosis patients, (incl.)- lesions unmasked, (excl.)- lesions masked out.

Correlation metric	Group	GM				WM			
		FA	MD	FA (skel.)	MD (skel.)	FA	MD	FA (skel.)	MD (skel.)
**Pearson, r_p**	**HC**	**0.31 (p<0.05)**	-0.0031	-0.07	0.025	**0.47 (p<0.001)**	-0.23	-0.17	0.0091
**Spearman, ρ**	**HC**	0.28	-0.032	-0.041	-0.0055	**0.48 (p<0.001)**	-0.25	-0.15	-0.066
**Huber slope**	**HC**	0.74	-6.7E-9	-0.0103	6.2E-7	0.51	-8.7E-06	-0.23	-1.4E-7
**LOO, r_{CV}**	**HC**	0.24	-0.94	-0.75	-0.94	0.44	-0.94	0.041	-0.95
**Pearson, r_p**	**MS (incl.)**	0.07	0.17	-0.037	0.019	**0.305 (p<0.05)**	**-0.24 (p<0.05)**	-0.081	0.067
**Spearman, ρ**	**MS (incl.)**	0.095	0.13	-0.035	-0.035	**0.28 (p<0.05)**	**-0.304 (p<0.05)**	-0.061	0.06
**Huber slope**	**MS (incl.)**	0.23	1.1E-5	-0.028	2.9E-6	0.35	-3E-5	-0.066	1.6E-5
**LOO, r_{CV}**	**MS (incl.)**	-0.054	-0.93	-0.53	-0.93	0.24	-0.93	-0.17	-0.93
**Pearson, r_p**	**MS (excl.)**	0.059	0.095	-0.036	0.0103	**0.39 (p<0.001)**	**-0.33 (p<0.001)**	-0.066	0.046
**Spearman, ρ**	**MS (excl.)**	0.085	0.082	-0.013	-0.05	**0.36 (p<0.001)**	**-0.38 (p<0.05)**	-0.056	0.058
**Huber slope**	**MS (excl.)**	0.23	6E-6	-0.024	5.3E-7	0.5	-4.6E-05	-0.052	1.2E-5
**LOO, r_{CV}**	**MS (excl.)**	-0.076	-0.94	-0.59	-0.94	0.34	-0.93	-0.25	-0.93

Table S4. Group mean fractional anisotropy (FA), mean diffusivity (MD) and fractal dimension (FD) values in the whole skeleton and in significant voxels obtained for different models in healthy controls (HC) and multiple sclerosis (MS) cohort obtained for sDTI. Covariates used in a given model are mentioned after the plus sign. All FA and MD values are significantly different between HC and MS (p<0.001). Effect size (Cohen's d) is also presented for the difference between HC and MS.

Metric	Model	n_{voxels}	HC	MS	Cohen's d
FD (whole skeleton)	box size: 1 – 2	--	1.812	1.823	--
	box size: 1 – 4	--	1.874	1.885	--
	box size: 1 – 8	--	1.953	1.954	--
	box size: 8 – 16	--	2.267	2.234	--
FA (whole skeleton)	MS vs. HC	85906	0.407 ± 0.016	0.383 ± 0.030	-0.94
MD (whole skeleton) (10^{-3} mm^2/s)	MS vs. HC	85906	0.673 ± 0.018	0.719 ± 0.045	1.25
FA (significant voxels)	MS vs. HC	66209	0.416 ± 0.017	0.387 ± 0.033	-1.04
	MS vs. HC + Age	64202	0.417 ± 0.039	0.388 ± 0.033	-1.04
	MS vs. HC + Age + SDMT	68280	0.415 ± 0.039	0.387 ± 0.033	-1.02
	MS vs. HC + Age + SDMT + EDSS	56354	0.427± 0.017	0.400 ± 0.034	-1.06
MD (significant voxels) (10^{-3} mm^2/s)	MS vs. HC	66209	0.681 ± 0.019	0.740 ± 0.048	1.28
	MS vs. HC + Age	64202	0.682 ± 0.019	0.733 ± 0.048	1.29
	MS vs. HC + Age + SDMT	68280	0.680 ± 0.018	0.730 ± 0.048	1.28
	MS vs. HC + Age + SDMT + EDSS	56354	0.682 ± 0.019	0.734 ± 0.049	1.3

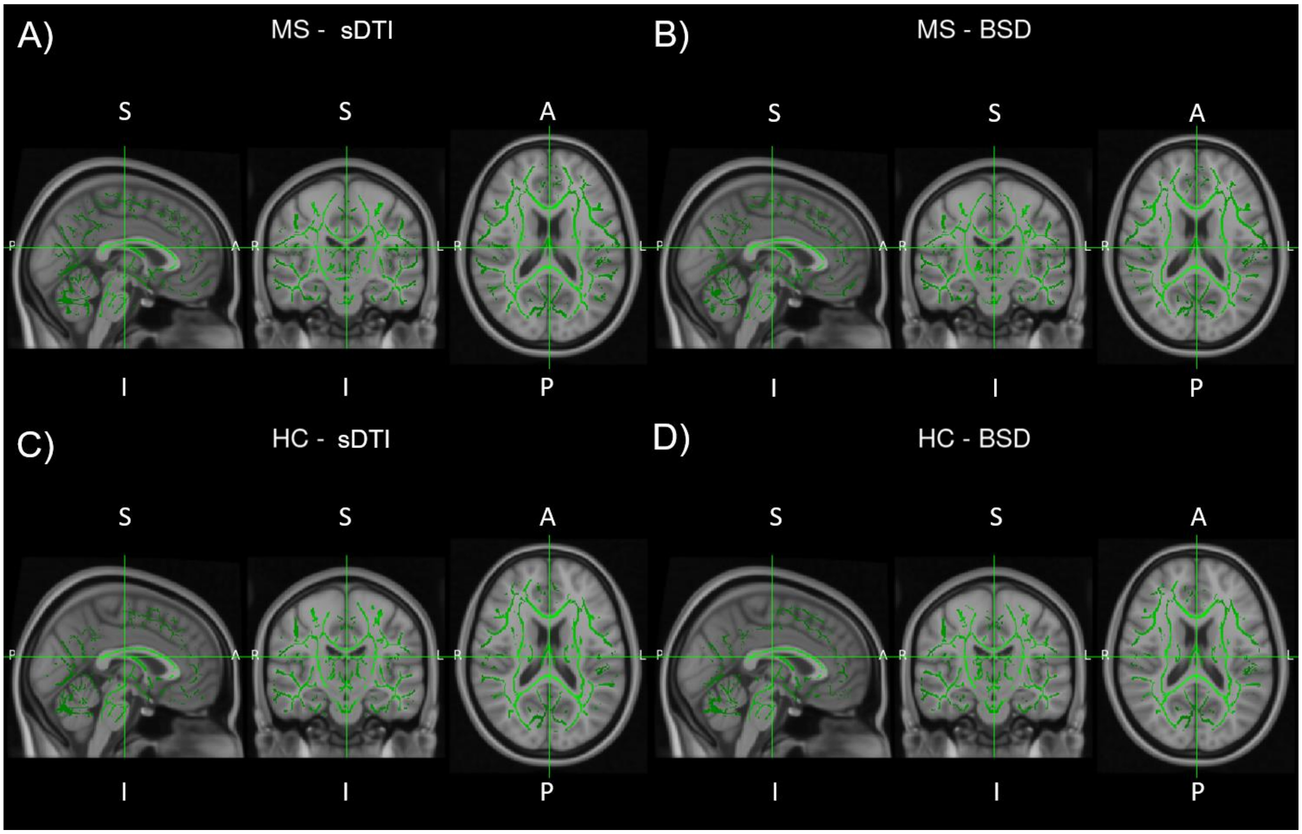

Figure S1. Skeletons from TBSS analysis in FSL overlaid on MNI152_T1_1mm image from FSL library for MS patients (upper row) and HC group (bottom row). Skeletons were obtained from FA map from diffusion tensor calculated using sDTI (A and C) and BSD approach (B and D).

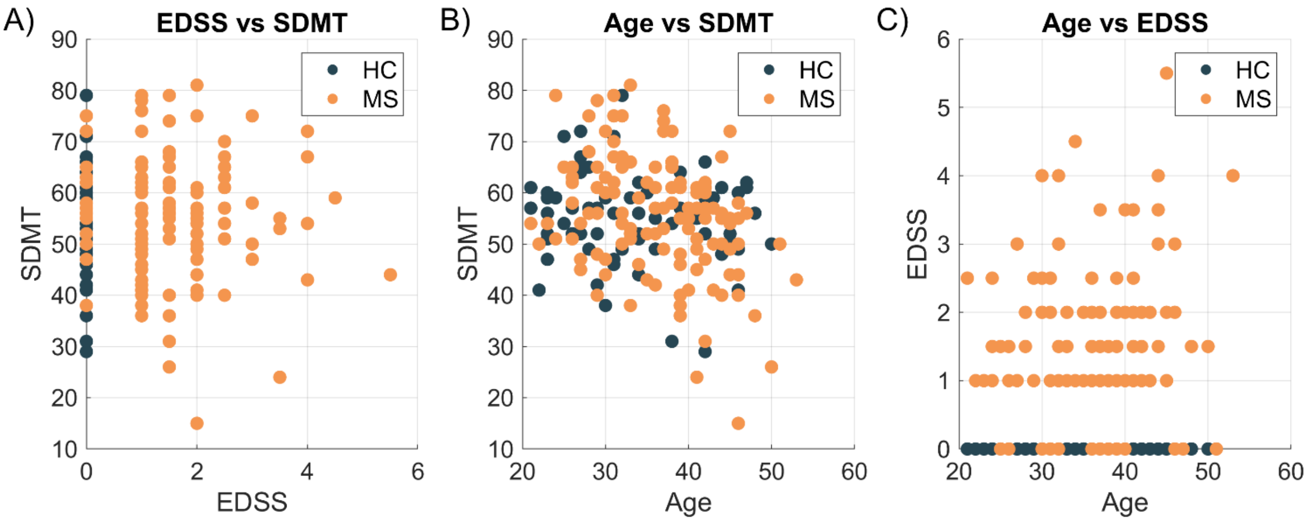

Figure S2. Correlation plots for covariates: Expanded Disability Status Scale (EDSS), Symbol Digit Modalities Test (SDMT), Age.

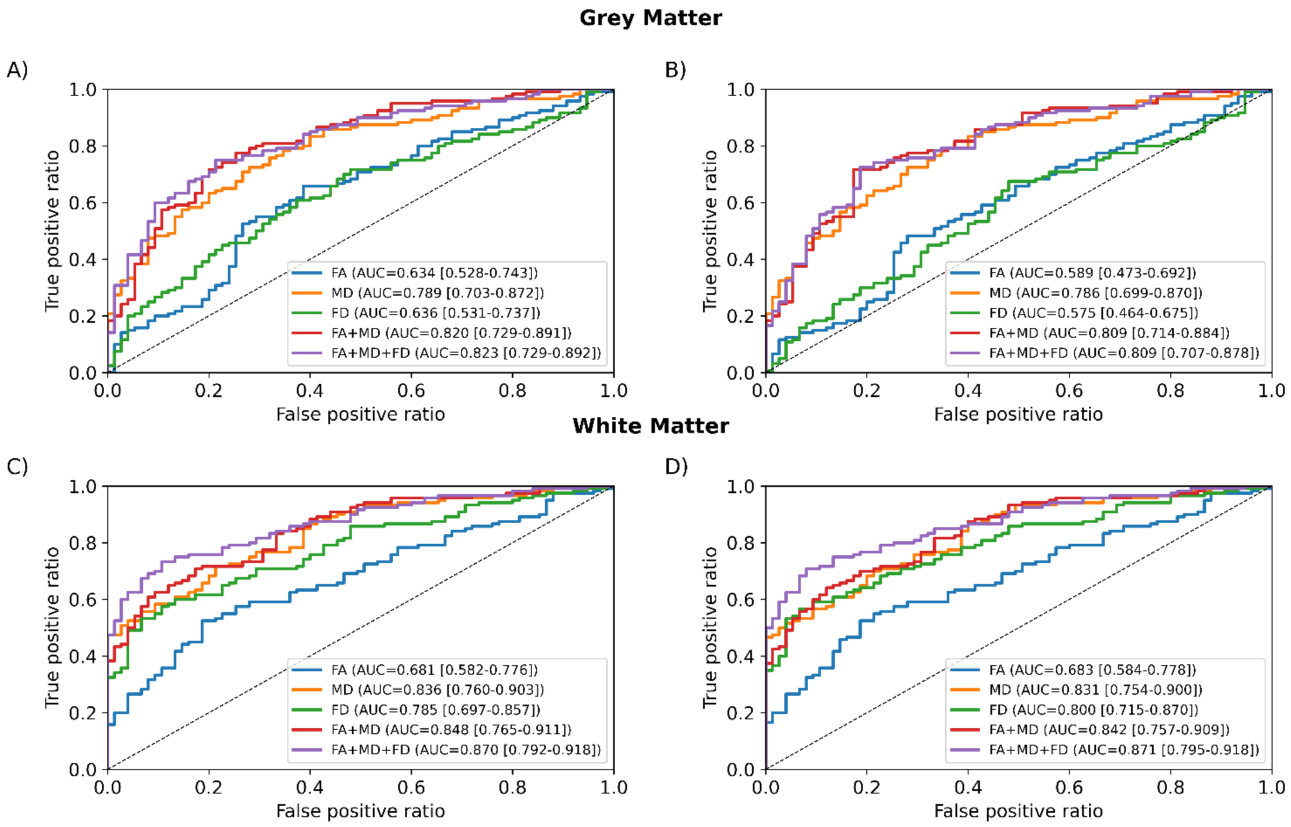

Figure S3. Receiver operating characteristic (ROC) curves obtained for sDTI approach.

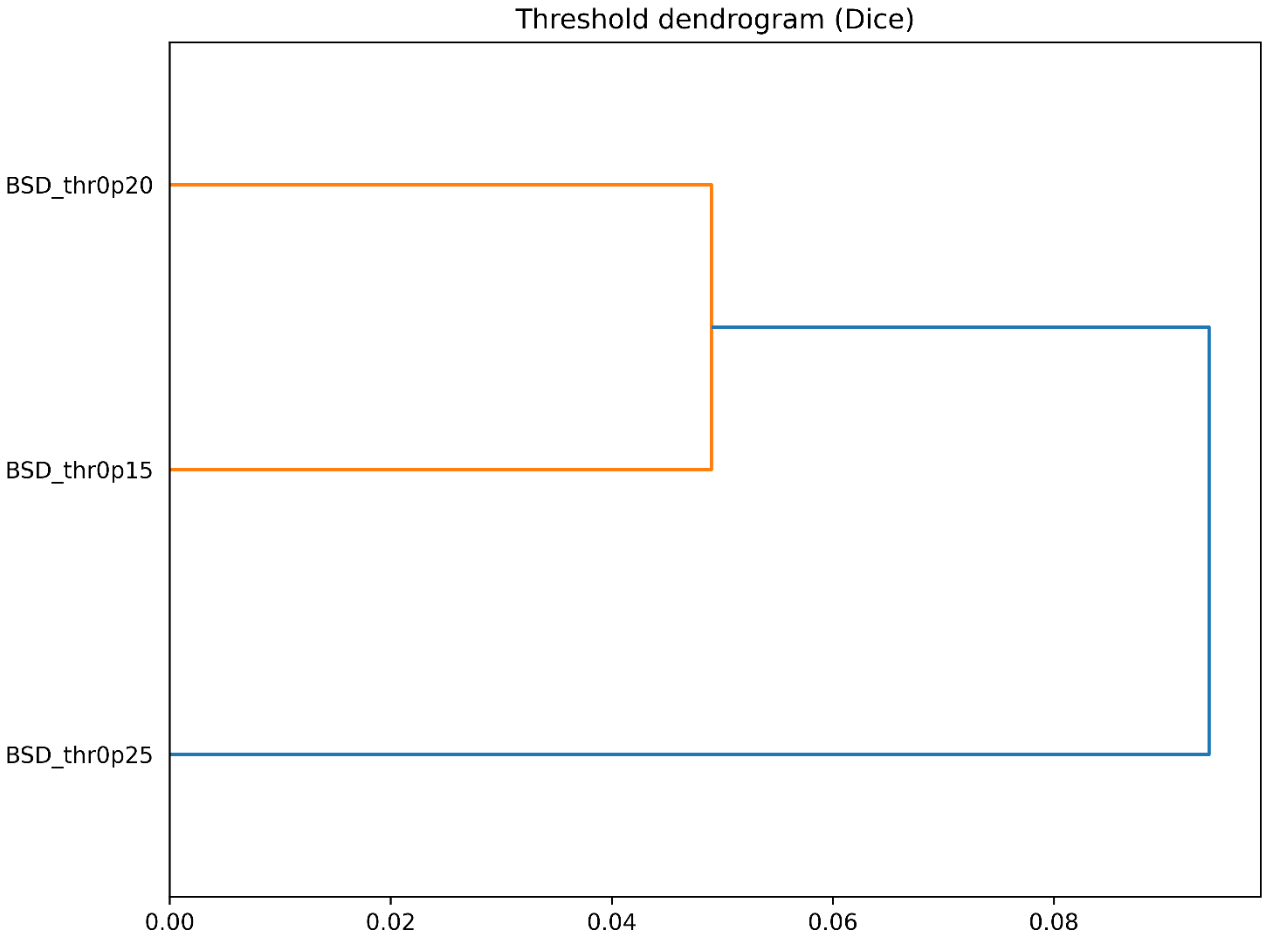

Figure S4. Dendrogram showing thresholds similarity based on Dice coefficient across models. X-axis shows 1-Dice value (shorter linkage = more similar).

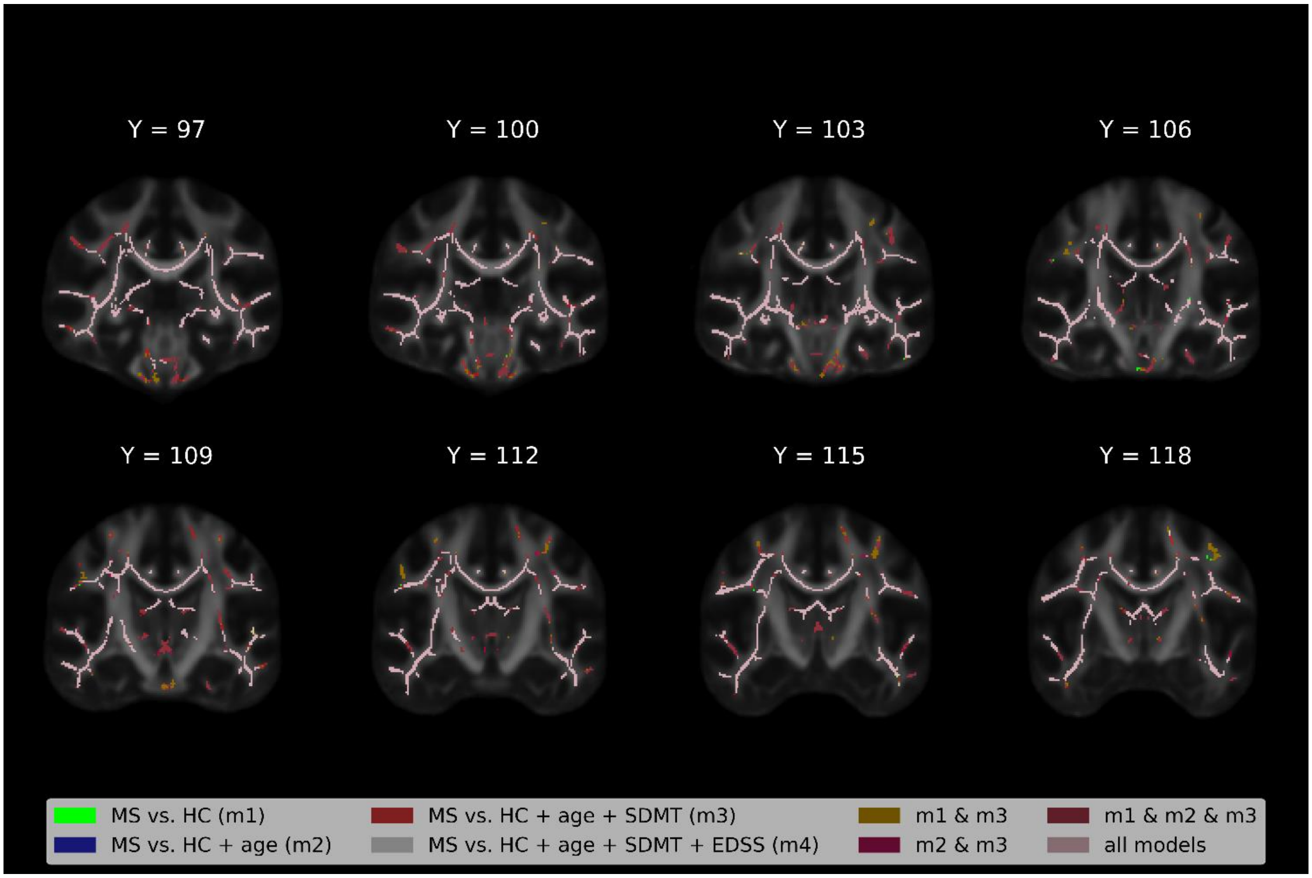

Figure S5. Example slices of significant voxels in FMRIB58 space obtained in a hierarchical model for fractional anisotropy (FA) variability.
